# GWAS to Identify Novel QTNs for WSCs Accumulation in Wheat Peduncle Under Different Water Regimes

**DOI:** 10.3389/fpls.2022.825687

**Published:** 2022-03-03

**Authors:** Arpit Gaur, Yogesh Jindal, Vikram Singh, Ratan Tiwari, Dinesh Kumar, Deepak Kaushik, Jogendra Singh, Sneh Narwal, Sarika Jaiswal, Mir Asif Iquebal, Ulavapp B. Angadi, Gyanendra Singh, Anil Rai, Gyanendra Pratap Singh, Sonia Sheoran

**Affiliations:** ^1^Department of Genetics and Plant Breeding, CCS Haryana Agricultural University, Hisar, India; ^2^ICAR-Indian Institute of Wheat and Barley Research, Karnal, India; ^3^ICAR-Indian Agricultural Statistics Research Institute, New Delhi, India; ^4^ICAR-Central Soil Salinity Research Institute, Karnal, India; ^5^ICAR-Indian Agricultural Research Institute, New Delhi, India

**Keywords:** water soluble carbohydrates, bread wheat, GWAS, QTN, gene prioritization, water stress

## Abstract

Water-soluble carbohydrates (WSCs) play a vital role in water stress avoidance and buffering wheat grain yield. However, the genetic architecture of stem WSCs’ accumulation is partially understood, and few candidate genes are known. This study utilizes the compressed mixed linear model-based genome wide association study (GWAS) and heuristic post GWAS analyses to identify causative quantitative trait nucleotides (QTNs) and candidate genes for stem WSCs’ content at 15 days after anthesis under different water regimes (irrigated, rainfed, and drought). Glucose, fructose, sucrose, fructans, total non-structural carbohydrates (the sum of individual sugars), total WSCs (anthrone based) quantified in the peduncle of 301 bread wheat genotypes under multiple environments (E01-E08) pertaining different water regimes, and 14,571 SNPs from “35K Axiom Wheat Breeders” Array were used for analysis. As a result, 570 significant nucleotide trait associations were identified on all chromosomes except for 4D, of which 163 were considered stable. A total of 112 quantitative trait nucleotide regions (QNRs) were identified of which 47 were presumable novel. QNRs *qWSC-3B.2* and *qWSC-7A.2* were identified as the hotspots. Post GWAS integration of multiple data resources prioritized 208 putative candidate genes delimited into 64 QNRs, which can be critical in understanding the genetic architecture of stem WSCs accumulation in wheat under optimum and water-stressed environments. At least 19 stable QTNs were found associated with 24 prioritized candidate genes. Clusters of fructans metabolic genes reported in the QNRs *qWSC-4A.2* and *qWSC-7A.2*. These genes can be utilized to bring an optimum combination of various fructans metabolic genes to improve the accumulation and remobilization of stem WSCs and water stress tolerance. These results will further strengthen wheat breeding programs targeting sustainable wheat production under limited water conditions.

## Introduction

Current photosynthates and the photosynthates reserved in various vegetative tissues are two primary carbon sources for developing grain in wheat. Current photosynthates are directly transferred to developing grains during optimal conditions when photosynthesis is undergoing. When photosynthesis is absent during the dark period or depressed due to senescence and/or hostile growth conditions, developing grains become more dependent on the redistributed reserved photosynthates for their carbon requirement (Schnyder, 1993). So, it can be hypothesized that genotypes with more reserved photosynthates are likely to yield more under various growth conditions. In wheat and other temperate cereal crops, these reserved photosynthates are famously known as water-soluble carbohydrates (WSCs), which accumulate mainly in stem and leaf sheath from stem elongation to the early grain filling phase and remobilize to grains in the later grain filling stage, which further depends on the genotypic potential and environmental factors (Dreccer et al., 2009; Blum, 2011). The highest amount of WSCs is stored in the peduncle and penultimate of the wheat stem (Wardlaw and Willenbrink, 1994). Glucose, fructose, sucrose, and fructans are primary WSCs in wheat plants, which redistribute as a significant carbon source under depressed photosynthesis. Hence, it is evident that WSCs are involved in the complex plant growth and development system under optimal and stressed conditions. Many previous studies have advocated the significant role of WSCs in coping with various abiotic and biotic stresses. For instance, sucrose signaling pathways leading to fructans and anthocyanin accumulation play a significant role in abiotic and biotic stress (Van den Ende and El-Esawe, 2014). The role of WSCs in cold acclimatization and freezing has also been advocated by Livingston et al. (2006). During water stress, fructans protect the plants by maintaining membrane stability and reducing osmotic potential (Valluru and Van Den Ende, 2008; Livingston et al., 2009). The role of fructans in reactive oxygen species (ROS) scavenging mechanisms and phloem-mobile signaling has been proposed in plants under water-stressed conditions (Peshev et al., 2013; Van den Ende, 2013).

In wheat, remobilization of WSCs to developing grains may potentially contribute to 10-20% and 30-70% of total grain weight under well-watered and water-stressed conditions, respectively (Goggin and Setter, 2004; Rebetzke et al., 2008; Ovenden et al., 2017a). Previous studies have supported the increased amount of stem WSCs and their remobilization in drought-tolerant wheat genotypes under water-stressed conditions (Yáñez et al., 2017; Ram et al., 2018; Li et al., 2020). Indeed, under well-watered and water-stressed conditions, differentially expressed genes (DEGs) affecting the accumulation and remobilization of WSCs have been reported in drought-sensitive and tolerant wheat genotypes (Xue et al., 2008; Zhang et al., 2009; McIntyre et al., 2011; Yáñez et al., 2017). The 1-*FEH* (Zhang et al., 2009), 6-*FEH* (Van Riet et al., 2006), 1-*SST* and 6-*SFT* (Kawakami et al., 2005), and Ta-*1FFT* and Ta-*6SFT* genes (Huynh et al., 2008, 2012) have been cloned and identified as major responsible genes for WSCs metabolism in wheat; however, the important role of sugar transporter genes like *TaSUT* (Ahmed et al., 2018) and MYB gene *TaMYb* (Xue et al., 2011) has also been described earlier. Nevertheless, the correlation between wheat stem WSCs and final grain yield is still unclear as some studies advocate a positive correlation between these two (Xue et al., 2008; Gao et al., 2017), and some others demonstrate nonsignificant even negative correlations (del Pozo et al., 2016; Ovenden et al., 2017a). Studies have suggested that the recent progress in yield potential of wheat in Australia, the United Kingdom, and southern Yellow and Huai Valley of China is somehow due to intentional or unintentional selection for increased WSCs (van Herwaarden and Richards, 2002; Shearman et al., 2005; Gao et al., 2017). Therefore, improvement for WSCs in wheat can be valuable for improving grain yield under favorable and unfavorable growth conditions. And it can also help in enhancing water-stress tolerance in future wheat.

Variations among wheat genotypes for accumulation and remobilization of WSCs lay within the differential capacity of photosynthesis, respiration, and carbon use efficiency of individual genotypes (Tricker et al., 2018). Many studies have shown substantial genetic variations for stem WSCs under various growth conditions and environments in wheat (Ruuska et al., 2006; Rebetzke et al., 2007; Dong et al., 2016; Fu et al., 2020). These variations are very sensitive to the environment (Ruuska et al., 2006). Yang et al. (2007) observed that quantitative trait loci (QTLs) associated with the accumulation and redistribution of WSCs may have different expression patterns at various stages of growth and/or in different environments. Therefore, the need of identifying more stable markers for WSCs is suggested by Zhang et al. (2014). Several QTL-mapping studies have been conducted to dissect the complex genetic architecture of WSCs in wheat at different growth stages and environments (Snape et al., 2007; Yang et al., 2007; Rebetzke et al., 2008; McIntyre et al., 2012; Li et al., 2020). Nevertheless, these studies produced limited knowledge about the genetic architecture of WSCs in wheat as bi-parental mapping populations have limited genetic diversity and recombination events. However, the foundation laid by these QTL mapping studies cannot be denied. Genome-wide association studies (GWAS) help overcome the above-said drawbacks of bi-parental QTL mapping and improve the resolution to get precise and deeper insights into genetic aspects of any trait. Only seven GWAS for the accumulation of stem WSC at different growth stages and environments have been conducted to the best of our knowledge. The first GWAS for accumulation of WSC at flowering, mid-grain filling, and maturity in wheat was carried out by Zhang et al. (2014) and Li et al. (2015) using 209 simple sequence repeat (SSR) markers. Dong et al. (2016) carried out GWAS for WSC accumulation at 14DAA in wheat stems by using a 90K SNP array (Wang et al., 2014). Since then, only three GWAS for understanding the genetic makeup of WSC in the wheat stem have been conducted (Ovenden et al., 2017b; Fu et al., 2020; Guerra et al., 2021).

GWAS and QTL mapping studies on WSC in wheat have contributed to decipher the various genetic aspects of this trait. However, the number of these studies is few. Moreover, none covered the individual components of stem WSCs, *viz*. glucose, sucrose, fructose, and fructans. Based on the knowledge of stem WSC as a trait, this study covers the individual components of WSC as: (1) final content or concentration of total WSC depends on the initial content/concentration of each component, (2) fructans, which make nearly eighty per cent of the total stem WSC, are derived from the polymerization of sucrose, (3) the amount of glucose, fructose, and sucrose triggers the hydrolysis of fructans to hexose and monosaccharides, which later becomes the source of carbon to developing grains. Presumably, this is the first report on GWAS that aimed to unveil the genetic architecture of accumulation of WSC and its major components 15 days after anthesis (15DAA) under three water regimes (irrigated, rainfed, and drought).

## Materials and Methods

### Plant Material and Field Experiments

An IIWBR Wheat Association Mapping Panel (IWAMP) was developed with a total of 301 bread wheat (*Triticum aestivum* L.) genotypes selected based on the pedigree information and Shanon index from a hefty germplasm pool of seven thousand lines (Supplementary Table 1) (Sheoran et al., 2019). This IWAMP was evaluated for two consecutive cropping seasons, *Rabi* 2017-18 and *Rabi* 2018-19, under three water regimes [irrigated (IR), rainfed (RF), and complete drought (DT)] at two locations: Chaudhary Charan Singh Haryana Agricultural University (CCS HAU) Hisar (29°09′N 75°42′E) and ICAR-Indian Institute of Wheat and Barley (ICAR-IIWBR) Karnal (29.686°N 76.989°E). The Hisar location represents the hot typic arid subeco region 2.3 of India, and Karnal represents semiarid eco-subregion 4.1 (Mandal et al., 2014). Trials of IR were carried out at both locations for both cropping seasons, whereas trials of RF and DT were conducted at Hisar and Karnal, respectively. For IR, four pre-anthesis and two post-anthesis irrigations were given, whereas irrigations were entirely ceased for RF and DT. For DT, genotypes were raised under rainout shelters that only cover the whole cropping area on a rainy day. All the trails were established in an alpha lattice design consisting of two replications. Each genotype was hand sown using IIWBR-Dibbler to ensure maximum homogeneity among trials (Sharma et al., 2016). Henceforth, a total of 8 environments and pooled-over water-managements namely E1: Hisar-Irrigated-2017-18; E2: Hisar-Rainfed-2017-18; E3: Karnal-Irrigated-2017-18; E4: Karnal-Drought-2017-18; E5: Hisar-Irrigated-2018-19; E6: Hisar-Rainfed-2018-19; E7: Karnal-Irrigated-2018-19; E8: Karnal-Drought-2018-19; IR: BLUEs (pooling the environments of irrigated water management); RF: BLUEs (pooling the environments of rainfed management); DT: BLUEs (pooling the environments of complete drought management) were used for association analysis.

### Phenotyping

Glucose, fructose, sucrose, fructans, and total WSC were assayed in each genotype’s peduncle (the first internode to spike base) under all the environments using Fourier-transformed near spectroscopy (FT-NIR). Briefly, from ten randomly chosen plants from each plot, peduncles of the main culm were sampled at 15DAA by cutting with a sharp knife just above the first internode and near the base of the spike, followed by immediate removal of leaf blades. Samples were dried for 60 min in a pre-heated (100°C) dry-air oven, followed by drying at 60°C for 48 h. Furthermore, each sample was chopped into small pieces of 1-2 mm. Finally, the whole sample was used for FT-NIR spectra acquisition. The in-house partial least square regression models (PLS-R), which are in line with Wang et al. (2011), were then used to quantify targeted traits (unpublished data). All PLS-R models were highly reliable with their high coefficient of determination (R^2^).

### Statistical Analysis of Phenotypic Data

For the present study, location × year × water regime was considered as an environment. Following mixed linear models (MLM) fitted with restricted maximum likelihood (REML) methods were used to create a type III ANOVA table with Satterthwaite’s method for the eight individual environments and environments pooled across three water regimes in R-studio using the package “*lmerTest*” (Kuznetsova et al., 2017), and the best linear unbiased estimators (BLUE) for each line were subsequently calculated:


Individual←lmer(dataset,trait∼Genotype+Replication+Block+(1|Replication:Block),REML=TRUE)

Pooled←lmer(dataset,trait∼Genotype+Replication+Environment+(Genotype:Environment)+(1|Block)+(1|Rep:Block:Environment),REML=TRUE)

in the above models “(1| X)” indicates a random intercept of the source of variation with a fixed mean.

Broad-sense heritability (*h*^2^) was calculated from the obtained variances using the following equations and was expressed in percentage:


$$\left(a\right)\mathrm{Individual}:\frac{\sigma_{g}^{2}}{\sigma_{g}^{2}+\frac{\sigma_{e}^{2}}{r}}\times 100$$

$$\left(b\right)\mathrm{Pooled}:\frac{\sigma_{g}^{2}}{\sigma_{g}^{2}+\frac{\sigma_{gy}^{2}}{y}\frac{\sigma_{e}^{2}}{\left(y\times r\right)}}\times 100$$

where the $\sigma_{g}^{2}$, $\sigma_{e}^{2}$, and $\sigma_{gy}^{2}$, are genetic variance, residual variance, and variance due to interaction between genotype and environment, respectively. The “*y*” and “*r*” are the total number of environments and replications, respectively.

### Genotyping

Genomic DNA was extracted from fresh leaves of 21-day-old seedlings using the CTAB method (Murray and Thompson, 1980). Genotyping was done using a 35K Axiom Wheat Breeders’ Array (Affymetrix UK Ltd., United Kingdom). SNP calling and filtering were done by PLINK (Purcell et al., 2007). Markers with missing values (GENO) more than 1%, minor allele frequency (MAF) less than 5%, and individuals with more than 10% missing SNP calls were rejected. The markers obtained were then ordered according to the genetic map available at CerealDb (Allen et al., 2017).

### Genetic Diversity, Population Structure, and Linkage Disequilibrium

Genetic diversity (*H*) and the polymorphism information content (PIC) for extracted SNP markers were calculated with PowerMarker v3.2.5 (Liu and Muse, 2005). A population structure based on a Bayesian algorithm was inferred in STRUCTURE software v2.3.4 (Hubisz et al., 2009) using a *K* value between 1 and 10. Three independent iterations for each K value were carried out with 10^5^ burn-in periods and Markov Chain Monte Carlo (MCMC) iterations. Optimal sub-population was identified using *ad hoc* statistics with ΔK (Evanno et al., 2005), calculated using the STRUCTURE HARVESTER tool (Earl and vonHoldt, 2012). Additionally, population stratification was validated using Principal Component Analysis (PCA) and *p*-value-based neighbor-joining (NJ) cluster analysis as performed with R-based GAPIT v2.0 (Tang et al., 2016) and MEGA-X (Kumar et al., 2018b), respectively. A marker-based VanRaden Kinship (VanRaden, 2008) (K-matrix) was also generated between all the 301 genotypes using GAPT-R v2.0 (Tang et al., 2016). Linkage disequilibrium (LD) was calculated separately for A, B, and D sub-genomes and the whole genome using PLINK software (Purcell et al., 2007). Later, the pattern of LD decay was estimated by plotting correlated pairwise *r*^2^ values against genetic distance with LOESS smoothening in R-studio using the “*loess()*” function. In the whole genome LD decay, a genetic distance at which the LOESS curve first touches the baseline *r*^2^ of 0.1 was considered the putative QTL-confidence interval (QTL-CI).

### Nucleotide-Trait Association

A compressed mixed linear model (CMLM) was implicated in GAPIT-R v2.0 (Tang et al., 2016) to estimate the association between polymorphic loci and BLUE values of each trait observed under an individual environment and estimated across different water regimes. To avoid any spurious effect produced by population structure, the K-PC model (Zhao et al., 2007) was adopted in which the first three principal components (PC) were used as covariates together with kinship information (K-matrix). *P-values* thresholds were determined by adjusting the false discovery rate (FDR) to 10%. A nucleotide-trait association (NTA) with threshold -*log_10_ p >* 3.0 was declared significant. Quantile-Quantile and Manhattan plots were recreated with the CMplot-R package in R-studio.

Each significant SNP was called a quantitative trait nucleotide (QTN), and the region within the genome-wide attenuation distance was called a quantitative nucleotide region (QNR). Each QNR is called “q” followed by the abbreviation of the trait (in the capital), a hyphen, a chromosome, a period, and a sequence in Arabic numbers (e.g., *qWSC-1A.1*).

### Quest for Putative Candidate Genes

Physical positions of significant SNPs in IWGSC *RefSeq*. v1.1 were retrieved from the CerealsDB (Wilkinson et al., 2020). Candidate genes within the 1-Mbp window of physically localized SNPs were retrieved from the EnsemblPlants database using the *biomartr* package of R, along with their genomic summary, InterPro, and GO term details. For *in silico* expression analysis, Affymetrix data of GSE9767 (Xue et al., 2008) and GSE87325 (Kumar et al., 2018a) were retrieved from NCBI’s Gene Expression Omnibus (GEO) database. The CEL files were processed with MAS5 algorithm with “affiyo” in R studio to extract the normalized transcript per million (TPM). The TPM was converted to log_2_ values in the MSExcel 2019. Additionally, the Genevestigator tool was used for the mRNA-based *in silico* expression analysis of overlapping genes under drought and related perturbation. The list of candidate genes was narrowed down to high-priority candidate genes by evaluating them on three candidate gene prioritization approaches, *viz*. (1) knowledge-based gene prioritization (KGP), (2) differential expression-based gene prioritization (DEGP), and (3) co-regulatory network-based gene prioritization (CNGP). The detailed method of gene prioritization can be found in Supplementary Method 1.

## Results

### Phenotypic Variability

**FIGURE 1:**
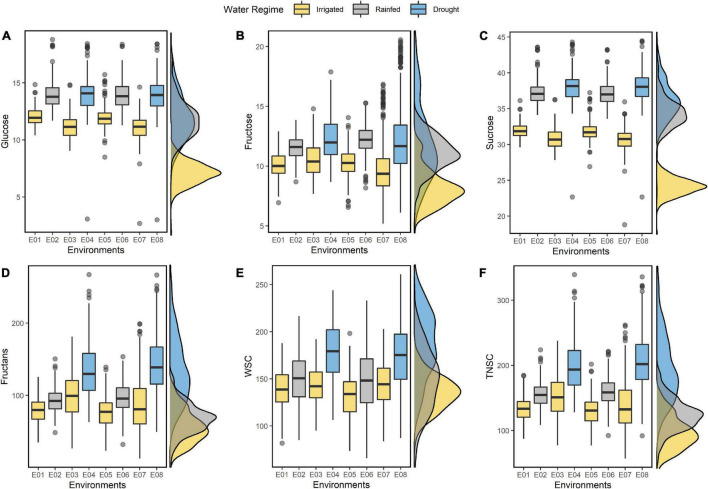
Boxplots summarizing the resulting BLUEs of glucose **(A)**, fructose **(B)**, sucrose **(C)**, fructans **(D)**, WSC **(E)**, and TNSC **(F)** estimated under eight different environments (E01-E08). On the left of each boxplot, the corresponding density plots show the distribution pattern of the traits with resulting BLUEs estimated by pooling of environments across corresponding water regimes.

The IWAMP carried continuous variation for all the studied traits under individual environments (E01-E08) and in the pooled data across three water regimes (Figure 1). Summary statistics on resulting BLUEs for individual environments and across three water regimes are detailed in Supplementary Table 2 and Table 1, respectively. Among all, fructans were predominating non-structural sugar, contributing 70 to 75 percent to total WSCs and TNSC. In contrast, the contribution of sucrose, glucose, and fructose was nearly 19-21, 7-8, and 6-7%, respectively. Values for all the soluble sugars were higher under stressed conditions than optimal conditions (Figure 1). An increment in all the studied traits showed a linear relationship with the degree of stress (Figure 1). Amount of glucose, fructose, sucrose, fructans, WSCs and TNSC under rainfed condition increased by 21, 17, 19, 9, 7, and 13%, respectively, and under drought stress 21, 21, 22, 61, 26, and 46%, respectively.

**TABLE 1:** A type-III ANOVA table with Satterthwaite’s method for a mixed linear model fitted by the restricted maximum likelihood method for the studied traits pooled across three different water regimes (irrigated, rainfed, and drought) and summary statistics.

Trait	WR	MSS_*G*_	MSS_*E*_	MSS_*G*×*E*_	Error	σ_*G*_	σ_*p*_	σ_*G*×*E*_	*h* ^2^	Min	Max	Mean	SE	CV	Sk	Ku
Glucose	Irrigated	2.11***	89.57***	1.60***	0.46	0.06	0.26	0.57	24.17	8.91	13.62	11.56	0.68	5.9	–0.08	2.47
	Rainfed	3.01***	0.03	2.44***	0.74	0.14	0.75	0.85	18.94	11.61	17.63	13.93	0.87	6.23	0.84	1.61
	Drought	4.96***	0.02	3.77***	0.97	0.3	1.24	1.4	23.99	8.79	17.75	13.98	0.99	7.06	–0.14	2.45
Fructose	Irrigated	5.77***	16.63***	4.37***	1.32	0.18	0.72	1.53	24.26	8.03	12.73	10.19	1.15	11.3	0.7	0.36
	Rainfed	2.73***	87.32***	2.24***	0.77	0.12	0.68	0.74	17.95	9.11	13.74	11.88	0.88	7.41	–0.2	0
	Drought	13.38***	0.01	10.75***	3.36	0.66	3.35	3.69	19.66	8	17.84	12.28	1.83	14.91	0.71	0.18
Sucrose	Irrigated	4.21***	126.07***	3.18***	1.11	0.13	0.53	1.04	24.47	27.52	34.29	31.32	1.05	3.36	–0.05	2.7
	Rainfed	5.81***	0.13	4.61***	1.64	0.3	1.45	1.48	20.65	34.06	42.11	37.18	1.29	3.46	0.88	1.63
	Drought	10.05***	1.06	7.55***	1.9	0.63	2.51	2.83	24.88	30.71	43.55	38.14	1.37	3.6	–0.09	2.28
Fructans	Irrigated	1950.00***	28818.00***	1302.00***	320.2	81	243.75	490.9	33.23	50.22	125.4	86.22	17.97	20.84	0.35	–0.42
	Rainfed	848.00***	1377.00**	488.00***	153.4	90	212	167.3	42.45	48.19	136.87	94.36	12.41	13.16	0.11	0.44
	Drought	3897.00***	14375.00***	1984.00***	517.06	478.25	974.25	733.47	49.09	77.74	237.98	139.12	22.69	16.31	0.63	0.02
WSC	Irrigated	1320.00***	13725.00***	844.00***	220.29	59.5	203.63	311.86	36.06	101.46	175.46	138.93	14.91	10.73	–0.04	–0.16
	Rainfed	2104.00***	4394.00**	1221.00***	336.2	220.75	526	442.4	41.97	84.76	214.91	148.74	18.24	12.26	–0.03	–0.29
	Drought	2360.00***	8123.00***	1382.00***	338.83	244.5	590	521.59	41.44	117.91	234.06	175.55	18.46	10.51	–0.15	–0.63
TNSC	Irrigated	2153.00***	22005.00***	1420.00***	324.56	91.625	559.25	547.72	34.05	102.92	181.97	139.29	11.69	8.39	0.35	–0.44
	Rainfed	1008.00***	2026.00**	565.00***	157.5	110.75	252	203.75	43.95	110.07	205.25	157.36	12.58	8	0.2	0.34
	Drought	4451.00***	15175.00***	2254.00***	524.68	549.25	1112.75	864.66	49.36	139.46	307.92	203.52	22.85	11.23	0.66	0.06

MSS_G_, The mean sum of squares of genotypes; MSS_E_, the mean sum of squares of environments; MSS_GXE_, the mean sum of squares of genotype x environment interaction; σ_G_, genotypic variance; σ_p_, phenotypic variance; σ_G×E_, genotype x environment variance; h^2^, broad-sense heritability; SE, standard error; CV, coefficient variation (%) between replications; Sk, skewness; Ku, kurtosis. Significance levels; ***p < 0.0001 and **p < 0.001.

The ANOVA for eight environments (Supplementary Table 2) and pooled across three water regimes (Table 1) indicated substantial phenotypic variability within IWAMP at *p <* 0.0001 for all the six traits. Pooled ANOVA further demonstrated a significant effect of genotype (G) x environment (E) interaction on all the studied traits at *p* < 0.0001. Each of the studied traits showed high environmental wise *h*^2^ (Supplementary Table 2), ranging between 63.18% (for sucrose under E05) and 86.78% (for TNSC under E04), whereas low-to-moderate pooled *h*^2^ (Table 1) varying from 17.95% (for fructose under RF) to 49.36% (for fructans and TNSC under DT) was estimated across three water regimes. Thus, the extent of genetic variability and differential response of accumulation of water-soluble sugars in the wheat peduncle of IWAMP to water availability was in favor to conduct the GWAS.

### Marker Coverage, Population Structure, and Linkage Disequilibrium

**FIGURE 2:**
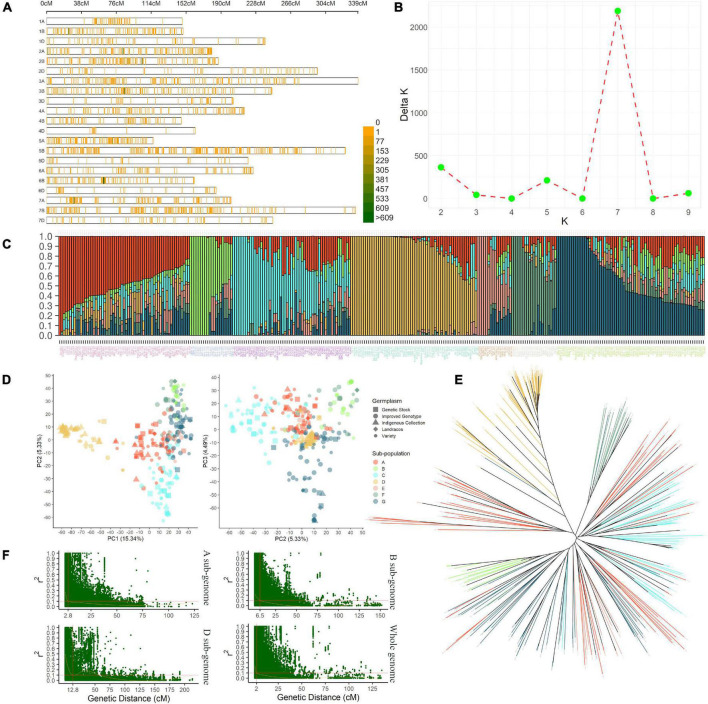
The density plot **(A)** shows the SNPs’ chromosome-wise distribution pattern with a 1 Mbp window in present IWAMP. The line graph **(B)** indicates the best *K* value estimated by the method described in Evanno et al. (2005) to identify the optimum number of sub-populations in the present AMP. The bar plot **(C)** summarizes the genetic composition of AMP; followed to this are PCA plots **(D)** and NJ cladogram **(E)**, which show a nearly similar pattern for population stratification. Finally, the scatter plots **(F)** show the genetic distance at which LD decayed for sub-genomes (A,B,D) and the whole genome.

A 35K SNP array-based genotyping of IWAMP produced 14,571 polymorphic SNPs after pre-processing of data. These markers covered a genome-wide genetic distance of 4,527 centimorgans (cM) with an average density of 0.31 cM (Figure 2A and Supplementary Table 3). About 5,728 SNPs were reported on sub-genome A, 7,357 on sub-genome B, and 1,486 on sub-genome D. The highest (1,360) and lowest (61) SNPs were reported on Chromosomes 2B and 4D, respectively. Furthermore, mean values for MAF, *H*, and PIC ranged from 0.16 (5D) to 0.32 (1D), 0.26 (5D) to 0.39 (1D) and 0.18 (5D) to 0.23 (5A), respectively. A summary of statistics generated from 14,751 polymorphic SNP markers is tabulated in Supplementary Table 3.

The stratification of IWAMP with STRUCTURE analysis defined seven subpopulations with substantial admixture (Figures 2B,C). The PCA explaining 25.16% with the first three PCs of the variation (Figure 2D) and an NJ-based cladogram (Figure 2E) showed fine consistency with population structure. The highest 69 genotypes clustered in sub-population “G” and the lowest 21 in sub-population “F.” The sub-population “G” was mainly composed of 32 improved genotypes (IG), which are ca. 36% of the total IG included in the present IWAMP. Contrastingly, sub-population “F” was a mixture of different germplasm types included in the present IWAMP. Interestingly, ca. 72% of the total indigenous collections (IC) clustered into sub-population “D,” whereas the rest were in sub-population “A,” “C,” and “G.” Based on decay in linkage disequilibrium (LD), the CI of a QNR was determined. At the cut-off value (*r*^2^ = 0.1), LD decayed at genetic distances 2.8 cM, 6.5 cM, and 12.8 cM for sub-genomes A, B, and D, respectively, and at 2 cM for the whole genome (Figure 2F).

### QTNs for Stem Water-Soluble Carbohydrates

**FIGURE 3:**
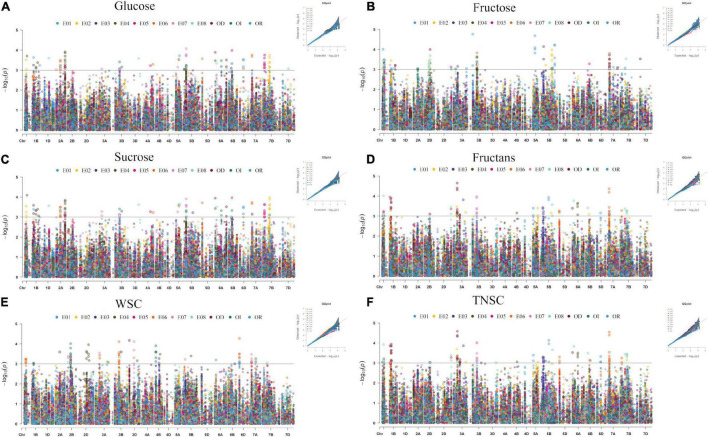
Manhattan and quantile-quantile plots summarizing the GWAS results for glucose **(A)**, fructose **(B)**, sucrose **(C)**, fructans **(D)**, TNSC **(E)**, and WSC **(F)** in IWAMP of 301 bread wheat genotypes evaluated under eight distinct environments. The *x*-axis and *y*-axis refer to chromosomes and *–log_10_*(*p*) values for different traits, and the colors of dots refer to different datasets.

In the existing GWAS, eleven phenotypic datasets consisting of eight environments (E01-E08), which received three different water regimes, *viz*. irrigation, rainfed, and severe drought and pooled over different water regimes, were used along with 14,571 high-quality SNP markers. The quantile-quantile plots (Figure 3) indicated that the compressed maximum likelihood method (CMLM) was the best model to identify significant quantitative trait nucleotides (QTNs) with adequate control on the false-positive rate. The present GWAS yielded 382 significant QTNs at *-log_10_ p* values ≥ 3.0 for the targeted traits across eleven datasets (Figure 3) on all chromosomes except 4D. The number of QTNs detected for different traits in eleven datasets was 28 (E01), 67 (E02), 31 (E03), 66 (E04), 40 (E05), 78 (E06), 41 (E07), 56 (E08), 54 (IR), 30 (RF), and 79 (DT). Unlike previous reports, we called an association between QTN and a trait as nucleotide-trait association(s) (NTAs). Thus, there were a total of 570 NTAs in the present GWAS (Table 2). The maximum NTAs were identified on sub-genome B (191) and individual chromosome 5B (42). Dataset E08 and E01 produced the highest (33) and the lowest (14) NTAs in the present GWAS. In different datasets, there were a total of 86, 101, 88, 93, 113, and 89 NTAs for glucose, fructose, sucrose, fructans, TNSC, and WSC, respectively. Comparatively, there were more NTAs for fructans, TNSC, and WSC under water-stressed conditions and for glucose, fructose, and sucrose under irrigated conditions.

**TABLE 2:** Number of NTAs (QNRs) identified in the present GWAS for WSC and its major components with eleven datasets.

Trait	NTAs	QNRs	Individual environments								Pooled		
			E01	E02	E03	E04	E05	E06	E07	E08	IR	RF	DT
Glucose	86	37	2 (1)	10 (4)	2 (2)	17 (8)	11 (5)	11 (6)	7 (4)	5 (5)	8 (6)	6 (4)	7 (3)
Fructose	101	39	14 (7)	11 (7)	5 (4)	13 (3)	5 (3)	7 (2)	1 (1)	11 (5)	14 (5)	8 (7)	12 (5)
Sucrose	88	36	3 (2)	11 (3)	3 (2)	13 (6)	11 (5)	11 (5)	9 (5)	3 (3)	10 (7)	5 (3)	9 (5)
Fructans	93	39	4 (2)	16 (7)	6 (5)	8 (3)	5 (4)	11 (4)	7 (1)	16 (8)	–	1 (1)	19 (9)
TNSC	113	42	5 (2)	17 (6)	11 (7)	5 (3)	6 (5)	14(4)	7 (1)	18 (9)	2 (2)	4 (2)	24 (9)
WSC	89	32	–	2 (2)	4 (3)	10 (7)	2 (1)	24 (6)	10 (5)	3 (3)	20 (6)	6 (3)	8 (3)

There was no significant QTN for WSC under E01 environment and fructans with pooled BLUEs corresponding to irrigated conditions. The effect of significant QTNs and explained phenotypic variance (PVE%) ranged from −0.61 to 0.72 and 3.47 to 6.69%, −1.10 to 1.47 and 3.55 to 7.76%, −0.87 to 1.03 and 3.47 to 7.01%, −23.19 to 15.09 and 3.16 to 10.31%, −24.51 to 16.34 and 3.1 to 9.7%, and −15.63 to 13.89 and 4.55 to 7.15% for individual traits glucose, fructose, sucrose, fructans TNSC, and WSC, respectively.

NTA common across two or more enviroments and/or with BLUEs was considered stable. Thus, we identified a total of 121 QTNs producing 163 stable NTAs (Supplementary Table 4), corresponding to different water regimes for six targeted traits, *viz*. glucose (21), fructose (34), sucrose (24), fructans (20), TNSC (30), and WSC (34), which were located on all chromosomes except on Chromosomes 1D, 4A, 4D, and 5D.

**FIGURE 4:**
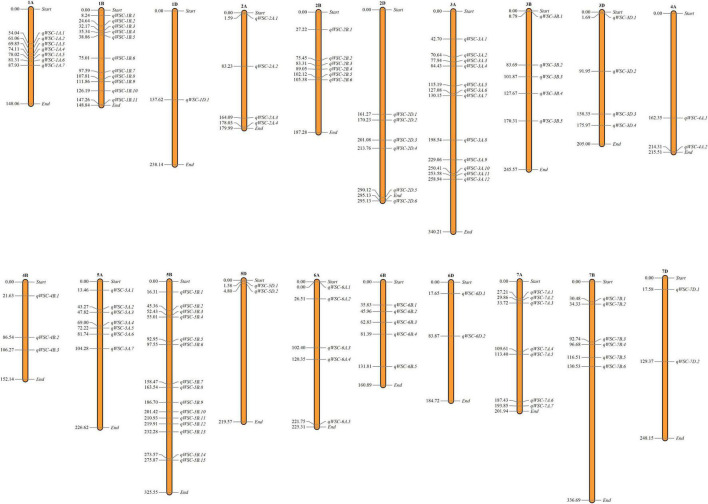
Dispersion of detected quantitative trait nucleotide regions (QNRs) across the bread wheat genome. The number on the left denotes the position of the first quantitative trait nucleotide (QTN) detected in the respective QNRs.

We used genome-wide LD attenuation distance (2 cM) to delimit detected QTNs into 112 genomic regions (Table 2 and Supplementary Table 4). These regions were designated as quantitative nucleotide regions (QNRs). The QNRs were unevenly dispersed across twenty wheat chromosomes (Figure 4). At most, fifteen QNRs were detected at Chromosome 5B and at least one at Chromosome 1D. Maximum (51) QNRs were dispersed within sub-genome B followed by A (44) and D (17). It indicated that sub-genome B is likely to be more critical for WSCs metabolism in bread wheat. The primitive sub-genome B is expected to inherit from *Aegilops speltoides*. The numbers of QNRs per trait were 37 (glucose), 39 (fructose), 36 (sucrose), 39 (fructans), 42 (TNSC), and 32 (WSC). We identified 25 QNRs that were consistent for two or more traits in more than one environment. A QNR, *qWSC-3B.2*, at physical position 3B:83.69-85.27cM, is likely to be a hotspot for WSCs metabolism since it carried 32 QTNs and was comprehensively associated with all the studied traits in different environments and water regimes. Another QNR, *qWSC-7A.2* at 7A:29.86-30.66 cM harboring 22 NTAs for fructose, sucrose, fructans, and TNSC, was likely to be a hotspot for WSC metabolism under water-stressed environments; however, within this genomic region, there were two QTNs for WSC content under E05 (irrigated conditions).

### QTN Localization and Genes in QNRs

**FIGURE 5:**
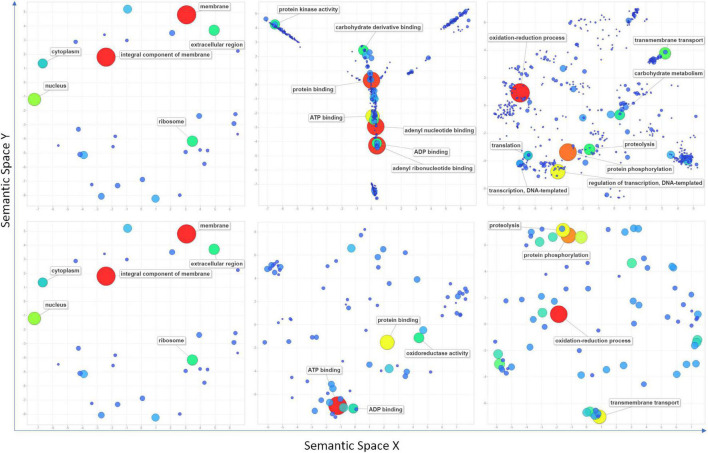
Scatter plots show cluster representatives of GO terms for cellular components, molecular functions, and biological processes (left to right) in semantic spaces for all the retrieved genes (above) within a 1 Mbp window from localized significant SNPs and overlapping genes (below). The size and colors of dots indicate the number of genes. Coordinates of each dot in semantic space are detailed in Supplementary Table 8. Terms with the highest values are mentioned in the figure.

High confidence genomic locations were identified for 209 QTNs distributed under 81 QNRs. A total of 3,496 high-confidence IWGSC genes were pulled out within the 1 Mbp window of the localized QTNs (Supplementary Table 5). One hundred fifty genes overlapped 161 QTNs, indicating that 48 QTNs were intergenic (Supplementary Table 6). Maximum 3 QTNs were found in gene *TraesCS7B02G446300* (7B: 709513978). Furthermore, nearly 1K genes were found in the 1-Mbp window of stable QTNs (Supplementary Table 7). All the genes retrieved were subjected to InterPro and gene ontology annotation using the EnsemblPlant and the AgriGO database in which InterPro domains and GO terms were retrieved for 2,939 and 2,501 genes, respectively. According to the ReviGO (Supek et al., 2011) summarization, these genes were engaged in 572 different biological processes in various cell components through their 480 molecular functions (Figure 5 and Supplementary Table 8). Maximum 203 genes were involved in the oxidation-reduction process followed by protein phosphorylation (173), regulation of transcription (127), transmembrane transport (79), proteolysis (70), and carbohydrate metabolism (60). Among overlapping genes, InterPro domains and GO terms were retrieved for 139 and 121 genes. Maximum 42 of overlapping genes were indulged in the oxidation-reduction process followed by protein phosphorylation (34), proteolysis (27), transmembrane transport (27), and translation (22) (Figure 5 and Supplementary Table 8).

We referred the public Affymetrix data of GSE9767 (Xue et al., 2008) and GSE87325 (Kumar et al., 2018a) for *in silico* expression analysis of genes retrieved within QNRs (Supplementary Table 5). There were 1,064 common genes with complete transcript information and valid results in both of the studies (Xue et al., 2008; Kumar et al., 2018a). In the peduncle of eight Seri/Bebax RILs contrasting to total stem WSCs at anthesis under rainfed environment, the average count of transcripts per million (tpm) ranged between 4.31 (*log*_2_ = 2.11; *TraesCS6D02G392300*) and 14151.17 (*log*_2_ = 13.79; *TraesCS1B02G317700*). In the latter experiment (Kumar et al., 2018a), the high-yielding drought-sensitive genotype WL-711 showed a range from 1.78 tpm (*log*_2_ = 0.83; *TraesCS3A02G149500*) to 161406.53 tpm (*log*_2_ = 17.30; *TraesCS1B02G317700*) in controlled environment and from 0.64 tpm (*log*_2_ = −0.64; *TraesCS7A02G482400*) to 153250.26 tpm (*log*_2_ = 17.23; *TraesCS1B02G317700*) in water stress environment. Likewise, the high-yielding drought-tolerant genotype C-306 carried a range between 1.30 tpm (*log*_2_ = 0.37; *TraesCS1A02G317311*) and 33444.93 tpm (*log*_2_ = 15.03; *TraesCS5B02G496000*) in controlled environment and 0.69 tpm (*log*_2_ = −0.54; *TraesCS7A02G482400*) to 32144.72 tpm (*log*_2_ = 14.97; *TraesCS3A02G405700*) in water stress environment. Additionally, a Genevestigator platform (Hruz et al., 2008)-based meta-transcriptomic analysis of 124 samples from various perturbation studies, including above two studies (control, water stress, heat, and heat + drought) suggested differential expression of all the overlapping genes (Supplementary Figure 1).

### Gene Prioritization

To prioritize putative candidate genes, we implicated three heuristic approaches, *viz*. KGP, DEGP, and CNGP. The first two approaches prioritized 1,372 and 312 genes in this study. In the latter approach, 331 genes were prioritized from eight co-regulatory networks (Supplementary Figure 2). Genes equally prioritized by all three approaches were called high-priority candidate genes (HPCGs), whereas genes prioritized by DEGP and CNGP were called low-priority candidate genes (LPCGs). Thus, in this study, we prioritized 64 HPCGs and 109 LPCGs. Additionally, 51 overlapping genes were found in either coregulatory network and called specially prioritized genes (SPCG). Furthermore, seven and nine SCGPs were also identified as HPCGs and LPCGs. Finally, 208 putative candidate genes delimited into 64 QNRs are prioritized in this study, which can be critical to understanding the genetic architecture of stem WSCs accumulation in wheat under optimum and water-stressed environments (Supplementary Table 9). Furthermore, we identified nineteen causative stable QTNs for 24 prioritized genes (Supplementary Table 10).

## Discussion

### The Variabilities

Substantial phenotypic variability was reported for studied traits. The high-phenotypic and GxE variance suggested environmental influence on the traits studied; however, the values for *h*^2^ indicted the possibilities for selection for these traits in breeding programs. Careful early selection of fructans, TNSC, and WSC may be possible due to their mild heritability (*h*^2^ = 30-60%), but, due to low heritability (*h*^2^ < 30%), glucose, fructose, and sucrose will require multiple selection cycles. Additionally, the high GxE interaction suggests that varietal development programs must be environment specific for these traits. These findings agreed with previous studies (Ruuska et al., 2006; Zhang et al., 2014; del Pozo et al., 2016). As Yáñez et al. (2017) put it, WSCs showed a tradeoff with soil moisture. In our study, maximum 49, 40, 31, 74, 60, and 50% and 33, 37, 27, 54, 42, and 39% increases in glucose, fructose, sucrose, fructans, TNSC, and, WSC, respectively, were recorded under dry and rainfed conditions. The present study followed the general pattern of stem WSCs accumulation, *i.e.*, drought-tolerant genotypes accumulate more stem WSCs in water stress. Indeed, genotypes that claim tolerance to heat stress, salinity stress, and a high degree of disease resistance also showed higher accumulation in water stress. However, drought-tolerant genotypes were more efficient in accumulating stem WSCs, which is likely due to the favorable conditions. Genotypes did not follow this pattern under irrigated trials. These results speculated the role of stem WSCs in drought stress and other stress conditions that must be further investigated.

Genotyping technique, marker coverage, and population structure are the determinating factors in GWAS, along with phenotypic variability. The present mapping panel was genotyped with Wheat Breeders’ Array (35K Axioms^®^ Array), which is an improved version of Axiom^®^ HD 820K wheat array and employed in previous studies (Alomari et al., 2017; Sheoran et al., 2019; Kumar et al., 2020). The number of polymorphic markers reported in the present study and their genome-wide coverage pattern agreed with the previous records (Berkman et al., 2013; Bajgain et al., 2015; Rahimi et al., 2019). As expected, roughly half of the total polymorphic SNPs mapped to B-genome followed by A (39%) and D (10%) genomes. For these polymorphic SNPs, genome-wide LD attenuated at 2 cM; however, for sub-genomes, the LD decayed at 2.8 cM (A-genome), 6.5 cM (B-genome), and 12.8 cM (D-genome). These values further presented the ample genetic diversity in our mapping panel. In addition, the LD decayed in the present study agreed with the previous studies (Ledesma-Ramírez et al., 2019; Hu et al., 2020).

The genetic population used in this study stratifies into seven distinct sub-populations (A to G). The genotypes clustered according to their pedigree or geographical origin. For instance, ten of the twenty members of the sub-population “B” had Pastor in their pedigree, and few others have “Bobwhite.” Interestingly, Pastor is the result of PFAU/SERI-82//(SIB)BOBWHITE (wheatpedigree.net). Likewise, in sub-population “D,” we can find a cluster of genotypes that are either collected from the Gujarat, Rajasthan, or Madhya Pradesh (MP) states of India or has been selected from the idigenous collection originated from these states. The substantial admixture further suggests that this population results from the extensive modern breeding program. On the other hand, most indigenous collections and some varieties (*e.g.*, Sonalika and HUW-598) were admixture less. These results indicate the broad genetic base of our study material and suitability for a successful GWAS.

### Comparison With Previous Studies

We have identified a substantial number of 382 QTNs delimited into 112 QNRs for the six targeted traits; many of which are endorsed by previous GWAS and bi-parental QTL mapping on stem WSCs. Forty-seven QNRs from the present GWAS overlapped with previously reported loci and genes for stem WSCs. These loci are likely to be the part of the same genomic region or loci (Supplementary Table 4). Presumably, our twenty novel QNRs carry 51 novel QTNs. Compared with previous studies, there were three intergenic QTNs (AX-94964616, AX-95114107, and AX-95169219), which Fu et al. (2020) also reported for stem WSC. In addition to this, five SNPs (AX-94428937, AX-94823847, AX-94474297, AX-110440726, and Ku_c56370_1155) from Fu et al. (2020) colocalized with our QTNs AX-94910470, AX-94708698, AX-94507107, and AX-94424668 on the same genes, *viz*. *TraesCS2B02G556600*, *TraesCS6A02G407700*, *TraesCS2A02G562500*, and *TraesCS3A02G301500*. Likewise, forty other QNRs overlapped the SNP markers identified by Fu et al. (2020). Two QTNs from *qWSC-1A.1* (1A:54.04 cM), namely AX-94583145 and AX-95137931 were just 0.4 cM and 0.54 cM away from the SNPs previously reported for stem WSCs (Dong et al., 2016; Ovenden et al., 2017b).

Seven of the previously cloned genes of WSC importance were found within the vicinity of QTNs identified in this GWAS. A sugar transporter gene, *TaSUT* (2A:733.56 Mbp) (Ahmed et al., 2018), was identified ca. 15 Mbp downstream from the QTN AX-94416062 of *qWSC-2A.3*, which was associated with sucrose content in plants under optimal conditions. Transcriptional factor *TaMYB13-1* (Xue et al., 2011) (1A:746.63 Mbp), which upregulates sucrose concentration and activates fructosyl transferase genes, was found within the vicinity of QTN AX-94838498 (3A: 743.75 Mbp) from *qWSC-3A.12*- controlling fructose content. QTN AX-94619716 (4A: 739.76 Mbp) from *qWSC-4A.2* was associated with glucose and sucrose content under water-stressed condition and had *6-SFT* (4A:739.06 Mbp), a gene responsible for β-2,6-linked fructans synthesis from sucrose (Kawakami and Yoshida, 2005), in its proximity. *1-FEH* gene hydrolyses β-2,1-linkages of fructans to break it into simpler sugars. It has been found directly correlated with sucrose, fructans, and WSC content in parts of the wheat stem, especially under water-stressed conditions (Van Den Ende et al., 2003; Yang et al., 2004). In our study, this gene colocalized with QTNs AX-94556117 and AX-94543047 of region *qWSC-6A.2* (6A:26.51 cM) controlling glucose, sucrose, fructans, TNSC, and WSC in water-stressed plants.

Furthermore, gene *TraesCS4A02G485400* (*6-SFT*) found in the neighborhood (0.69 Mbp) of QTN AX-94619716 (4A:739763001 bp). Earlier, Huynh et al. (2008) identified a potential QTL, namely *QGfc.aww-7A.1* (7A:2.2-8.2 cM), for grain fructans concentration in Berkut × Krichauff double-haploid lines, from which they further cloned a cluster of four fructans biosynthesis genes (Huynh et al., 2012). We BLAST the sequences of these genes against IWGSC *RefSeq1*.*1* and retrieved two genes with 100% similarity, *viz*. *TraesCS7A02G009200* (7A:4014510-4016977 bp) and *TraesCS7A02G009800* (7A:4430034-4430058 bp), which they designated as *Ta1-FFT* and *Ta6-SFT*, respectively. These two genes were within the 1-Mbp window of QTN AX-95231996 (7A:3982822:4627547 bp) associated with fructose content under rainfed conditions. The QTN region, namely *qWSC-7A.2* (7A:29.86-30.66 cM), to which these two genes assigned in this study, seems to be the same region. In the findings of Huynh et al. (2012), we found traces of inheritance of this QNR with favorable alleles from the CYMMIT line “Pastor.” Looking back into the pedigree information (Supplementary Table 1), at least 20 genotypes were directly developed by “Pastor” as one of the parents, and few others had it in their lineage. We closely correlated the pedigree information with the favorable allele analysis, which indicated that the genotypes with early CIMMYT breeding lines in their background accumulated more suitable combinations of favorable alleles. Thus, presumably, favorable alleles of stem WSCs are inherited by the CIMMYT breeding lines across the globe, for which inadvertent selections continued. Earlier, Rebetzke et al. (2009) also marked an increasing trend in the Western Australia wheat breeding program with the involvement of the CIMMYT lines. This finding needs a further detailed discussion with validatory reports that would be beyond the objectives of the present article.

### Complex Genetic Architecture Regulates the Accumulation of Soluble Sugars

**FIGURE 6:**
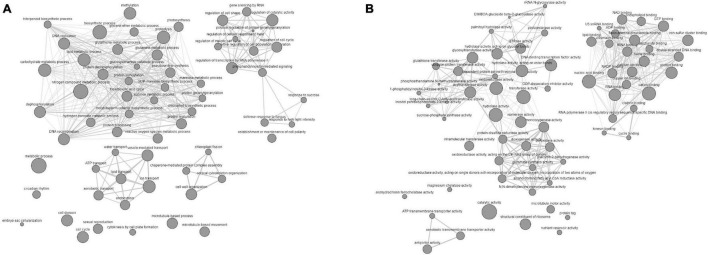
GO enrichment-based biological **(A)** and molecular **(B)** networks of hub genes indicating the involvement of various biological and functional groups in the accumulation of WSCs in the wheat stem under differential water conditions.

We observed that all the QTN regions were enriched with the genes, which may affect the carbohydrate metabolism and expression of studied traits in a C3 plant, depending upon the growth conditions. Furthermore, IPR and GO term enrichment analysis indicated that the genes with the molecular function of oxidation reduction, protein phosphorylation, and carrying genetic information overrepresented the defined QNRs (Figure 5). More specifically, the genes overlapping significant QTNs were located in the nucleus (100), chloroplast (30), mitochondria (12), cytoplasm (6), extracellular space (25), and various cellular membranes (Supplementary Table 8). Thus, they were likely to be responsible for the stem WSCs variation through other metabolic activities such as light-harvesting complex, electron transport system, sugar and ion transport, respiration, and many others. For instance, the QTN AX-94710575 associated with fructans and TNSC variation in water-stress condition tagged *Os01g0227100* orthologous chlorophyll-*b* reductase gene (*TraesCS3A02G151900*). The chlorophyll-*b* reductase initiates the process of reduction of chlorophyll-*b* to 7-hydroxymethyl chlorophyll-*a*. The downregulation or absence of chlorophyll-*b* reductase genes in *Arabidopsis* and *Oryza sativa* has been associated with stay-green traits (Sato et al., 2009; Nakajima et al., 2012). Thus, this gene is reasonably associated with a more extended accumulation period for stem WSCs. A lower transcript level has been reported for *TraesCS3A02G151900* in the drought-tolerant C-306 genotype in contrast to the drought-sensitive WL-711 genotype under water-stress conditions (Kumar et al., 2018a). Likewise, this gene showed higher expression in RILs with high WSC content in the peduncle under rainfed conditions than RILs with low WSC content (Xue et al., 2008). A PsbQ gene (*TraesCS2B02G395700*) overlapped the QTN AX-94491160 associated with fructose content under severe drought-stress environment E04. The PsbQ protein is a part of the photosystem-II oxygen-evolving complex and is required for the calcium ion retention that is crucial for the stability of photosystem-II (Sasi et al., 2018). The previous study on *Arabidopsis* has suggested that the long-term water stress markedly decreases the PsbQ protein, thereby affecting the functionality of photosystem and photosynthesis. In our research, the E04 environment represents the long-term drought condition. The *TraesCS3A02G425500*, a sucrose synthase/phosphatase-encoding gene, was tagged by a stable QTN AX-94501648 responsible for TNSC variation in our GWAS panel under severe drought stress. Increased expression of sucrose synthase genes is negatively correlated with total WSC content in the bread wheat peduncle under water-stressed conditions (Xue et al., 2008). A sugar transporter gene (*TraesCS4B02G371700)* overlapped the QTN AX-94619716 associated with glucose and sucrose content under a rainfed environment (E06). As revealed by Knetminer, the *TraesCS4B02G371700* has a direct physical relationship with the sucrose transporter gene (SUT4) that cooccurs with carbohydrate and sucrose content in bread wheat. Likewise, there were a total of 72 overlapping genes that had a direct or indirect connection with either or all the studied traits in Knetminers. About 26 stable QTNs were likely to be associated with 24 genes prioritized for stem WSCs metabolism in the present study (Supplementary Table 10). These genes were involved in a wide array of molecular functions and biological processes in wheat. For instance, AX-94865722 (2A:765292309 bp), a stable QTN associated with glucose and sucrose content under severe water-stress environment (E04 and DT), tagged two NAC-like genes (*TraesCS2A02G566000* and *TraesCS2A02G566100*). Two stable QTNs (AX-94995102 and AX-94495814) for fructans and TNSC content under severe water stress were found causative from a MYB-domain protein *TraesCS1B02G316200*. Earlier, Kooiker et al. (2013) demonstrated the role of MYB-domain protein in enhanced accumulation of fructans in bread wheat. The NAC genes affect carbohydrate metabolism, phytohormones, and transmembrane transporters-related genes, hence crucial for drought tolerance and reducing grain yield losses (Chung et al., 2018). Likewise, a sugar/inositol transporter gene (*TraesCS4B02G371700*) was tagged under optimum water conditions for total stem WSC content with a stable QTN AX-94546364 (4B:657172059 bp). The stable and novel QTN from novel QNR *qWSC-3A.7*, AX-94501648 (3A:667793607 bp), was significant for TNSC in severe drought, tagged *Os01g0919400*-like sucrose phosphate synthase gene *TraesCS3A02G425500*. Furthermore, the co-regulatory network (CRNs) study based on the publicly available Affymetrix data (GSE9767 and GSE87325) indicated that a complex network of many genes works behind the veil for regulating the accumulation of stem WSCs. We reported that the number and type of participatory genes significantly change with the genetic background and growth conditions at an individual level. The twelve CRNs allowed us to better narrow down to a few hub genes (301). No hub gene was reported common across the twelve CRNs. However, there were hub genes common between different genetic backgrounds. The functional enrichment analysis followed by functional group-based networking (Figures 6A,B) of hub genes left us with the information on the interaction of various functional groups interacting with major fructans metabolic genes, which we reported in QNRs *qWSC-4A.2* and *qWSC-7A.2*, and other sugar metabolic genes, in a complex and coordinated network. Figure 6A indicates various biological functions, such as chlorophyll biosynthesis, photosynthesis, and respiration, correlate with the metabolic process of WSCs.

### Prospects for Stem Water-Soluble Carbohydrates Improvement in the Present Study

Consistent with many previous studies, our results demonstrate several advantages, such as a more significant number of genotypes with substantial genetic variability at phenotypic and genetic levels, consideration of multiple environments pertaining to different water regimes, and consideration of individual components of water-soluble carbohydrates as a determining factor in final stem WSCs content. However, the present investigation carried the well-defined limitation of GWAS, which includes the inability to detect rare alleles and epistasis. In the current research, we have identified twenty novel QNRs responsible for the expression of studied traits.

With the keen observation of the results, we realized that the accumulation of stem WSCs is a low to moderately heritable and environmentally influenced trait. The complexity in improving this trait lies with the higher degree of genotype x environment (GxE) and QTN x environment (QnxE) interactions. Stability of QNRs over the QTNs across the environments indicated that the phenotypic expression of the studied traits is limited to the fewer genomic regions with a substantial number of genes bound within a single and complex regulatory network with a fewer number of hub genes that further modulate with the genetic background of an individual and external stimuli. Within 1.5Kbp upstream from the starting coordinates (bp) of the possible candidate and overlapping genes, we identified 130 different *cis*-regulatory elements (CREs) with the help of PlantCARE (Lescot, 2002). These CREs were responsive to various hormones (ABA, gibberellin, auxins), lux, temperature, water, and circadian rhythm, including those having tissue-specific expression (Supplementary Figure 3). Probably, this is why we detected a lesser number of stable QTNs and traces of environmental pleiotropy. Furthermore, the strong correlation among the studied traits is likely due to the stacking of genes rather than being controlled by a single gene.

**FIGURE 7:**
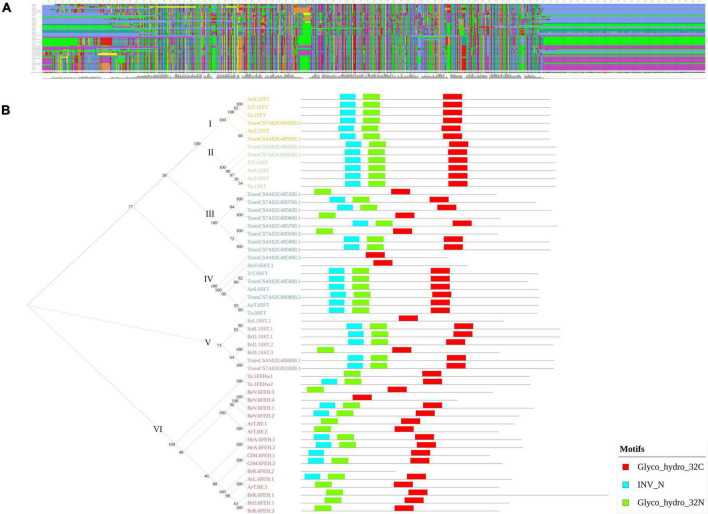
The multiple sequence alignment **(A)** and phylogram with a comparison between motif structures of the fructans metabolic genes of *Triticum aestivum* and model plant species **(B)**. In **(B)**, the Arabic numbers indicate the bootstrap values, whereas the Roman numbers indicate the cluster number.

Furthermore, the present study identified two genomic regions, *qWSC-4A.2* and *qWSC-7A.2*, with the clusters of fructans metabolic genes, two of which were the center of gene networks; however, their expression was subjected to genotype and water conditions. The genevestigator based *in silico* expression analysis of these genes showed differential expression, depending upon the pertubation (Supplementary Figure 4). Furthermore, these genes were subjected to phylogeny, evolutionary, and protein-modeling studies (Supplementary Method 2) for mining further opportunities. We found that these clusters carried multiple orthologs of fructosyl transferase genes translating into theoretically stable (except *TraesCS4A02G485000* and *TraesCS7A02G009100*) but structurally different proteins. The multiple sequence alignment showed many conserved amino acids across the fructans metabolic genes (Figure 7A). As put forward by Huynh et al. (2012), the phylogenetic analysis (Figure 7B) in this study indicated that fructosyl transferase genes (SST/SFT/FFT) have evolved from exohydrolase/invertase genes. Of the three different fructosyl genes, SFT type of genes is likely to evolve first, followed by SST and FFT. With MEME suite analysis, we identified three significant motifs of the glycosyl hydrolase family (INV_N, Glyco hydro 32N, and Glyco Hydro 32C) conserved across the fructans metabolic genes (Figure 7B). However, multiple sequence alignment of these motifs indicated that the substitutions of amino acids at few positions are likely to bring structural and functional changes. The Glyco hydro 32N and INV motifs are likely to carry a more significant number of conserved sites than the Glyco hydro 32C motif (Supplementary Figure 5). These substitutions are presumably located at active sites, bringing the difference in the functionality of FEH-, SFT-, SST-, and FFT-type genes despite the similar protein structure. Previously, Lammens et al. (2012) demonstrated that the major differences between functionality of various fructans metabolic genes lay within the few amino acid substitutions at active sites. Therefore, one type of the fructans metabolic gene can be converted to another type with the help of advanced gene-editing techniques like CRISPR to bring different fructans genes to an optimum combination that improves the accumulation and remobilization of stem WSCs. Earlier, in tobacco, Bie et al. (2012) demonstrated improved fructans biosynthesis and abiotic stress tolerance with the combinational transformation of wheat’s *1-FFT*, *6-SFT*, and *1-SST* genes. Additionally, 3D protein models (Supplementary Figure 6) were predicted with the deep learning methods (Wang and Xu, 2013; Ma et al., 2015) as applied in the RaptorX (Källberg et al., 2012). The validatory reports on the 3D models generated with Structure Validation Server (SAVESv6.0) indicated the high reliability of the predicated models (Supplementary Table 11).

## Conclusion

Finally, based on our results, we conclude that the final content of total WSC in stem depends on the initial partitioning of the individual sugars, *viz*. glucose, fructose, and sucrose and their conversion to fructans. Therefore, the accumulation of stem WSCs in wheat shall depend on the machinery affecting the metabolism of these sugars, which further strengthens with the identification of candidate genes involved in electron transport systems, photosynthesis, respiration, and signaling. Unfortunately, due to the presence of CREs, these genes are quite sensitive to external stimuli and, accordingly, changes their expression pattern. In the present study, we identified novel QTNs and QNRs, showing the positive pyramiding effect of a few representative QTNs on total stem WSCs. But, bringing improvement in total stem WSC content with the pyramiding of this large number of QTN and candidate genes further subjected to large GxE and QnxE interaction remains a big challenge, even with the advanced genomic selection algorithms. Fructan metabolic genes identified on chromosomes 4A and 7A can be used to enhance the fructans biosynthesis and remobilization for improving the drought tolerance. Thus, alongside identifying novel QTNs and QNRs, and narrowing the knowledge of the molecular and genetic basis of stem WSCs accumulation in different water conditions, the present investigation indicates vast opportunities to study CREs as a breeding target for environmentally influenced traits and gene editing in improving fructans metabolism.

## Data Availability Statement

The datasets presented in this study can be found in online repositories. The names of the repository/repositories and accession number(s) can be found in the article/Supplementary Material.

## Author Contributions

SS, AG, and YJ designed the study. SS, RT, and AG managed the trials at ICAR-IIWBR, Karnal. AG, YJ, VS, and DeK managed the trials at CCS HAU, Hisar. AG carried out phenotyping at Karnal and Hisar. SN guided in wet lab analysis. JS provided the FTNIR spectroscope facility. AG, SS, SJ, MI, and UA conducted GWAS. AG and SS conducted post GWAS analysis. AG and SS drafted the manuscript. YJ, SS, VS, GS, DK, GPS, and AR provided overall guidance and finalized the edited manuscript. All authors read and approved the final version of the manuscript.

## Conflict of Interest

The authors declare that the research was conducted in the absence of any commercial or financial relationships that could be construed as a potential conflict of interest.

## Publisher’s Note

All claims expressed in this article are solely those of the authors and do not necessarily represent those of their affiliated organizations, or those of the publisher, the editors and the reviewers. Any product that may be evaluated in this article, or claim that may be made by its manufacturer, is not guaranteed or endorsed by the publisher.
