# Supplementary material for: GWAS to Identify Novel QTNs for WSCs Accumulation in Wheat Peduncle Under Different Water Regimes
Source: Front Plant Sci. 2022 Mar 3;13:825687. doi: 10.3389/fpls.2022.825687 (PMC8928439; doi:10.3389/fpls.2022.825687)
Supplement: Supplementary file 1 [file Data_Sheet_1.PDF]

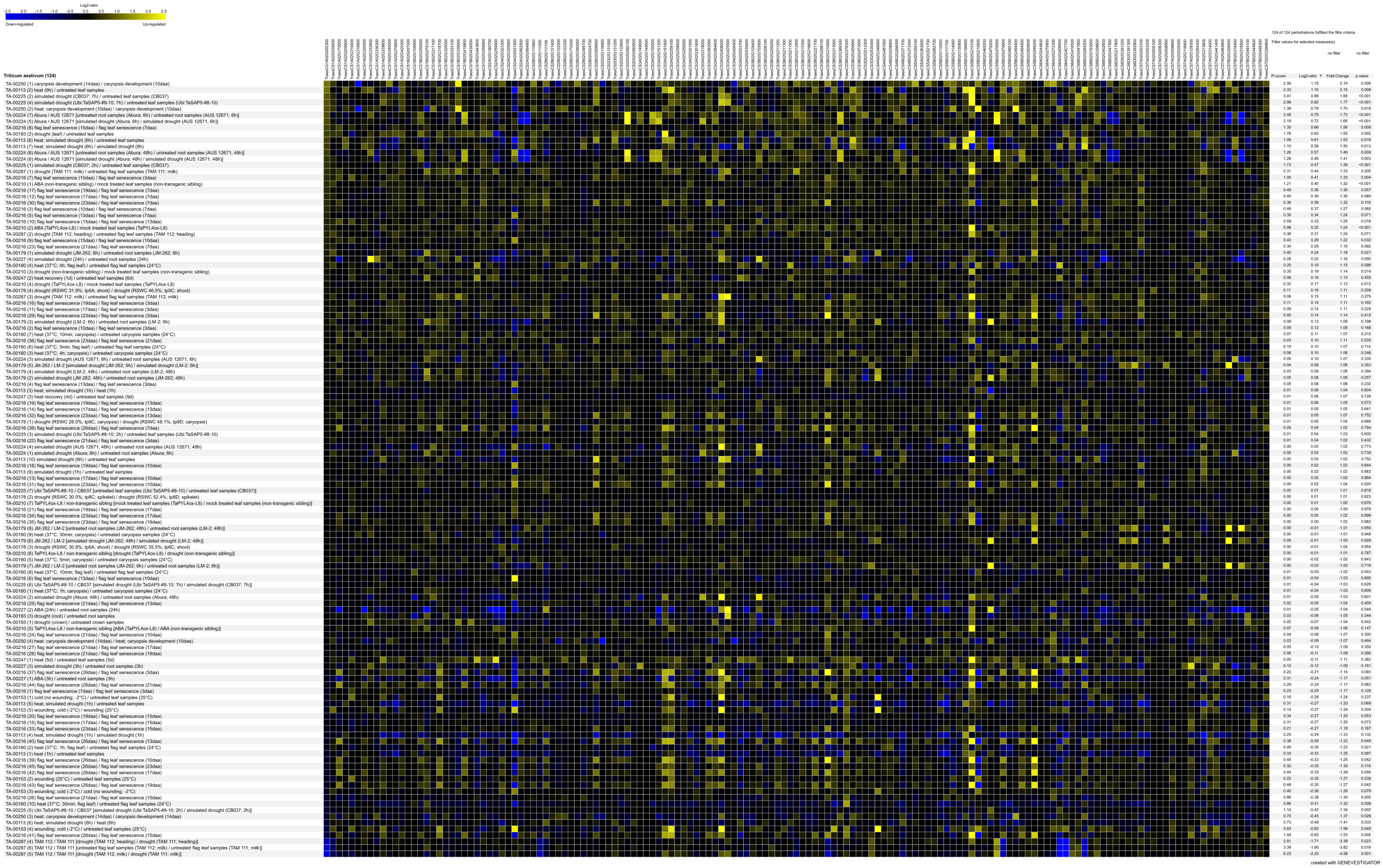

Figure S1: mRNA based differential expression of overlapping genes at 124 different drought and related perturbations in various samples of *Triticum aestivum* as retrieved from Genevestigator tool

(A)

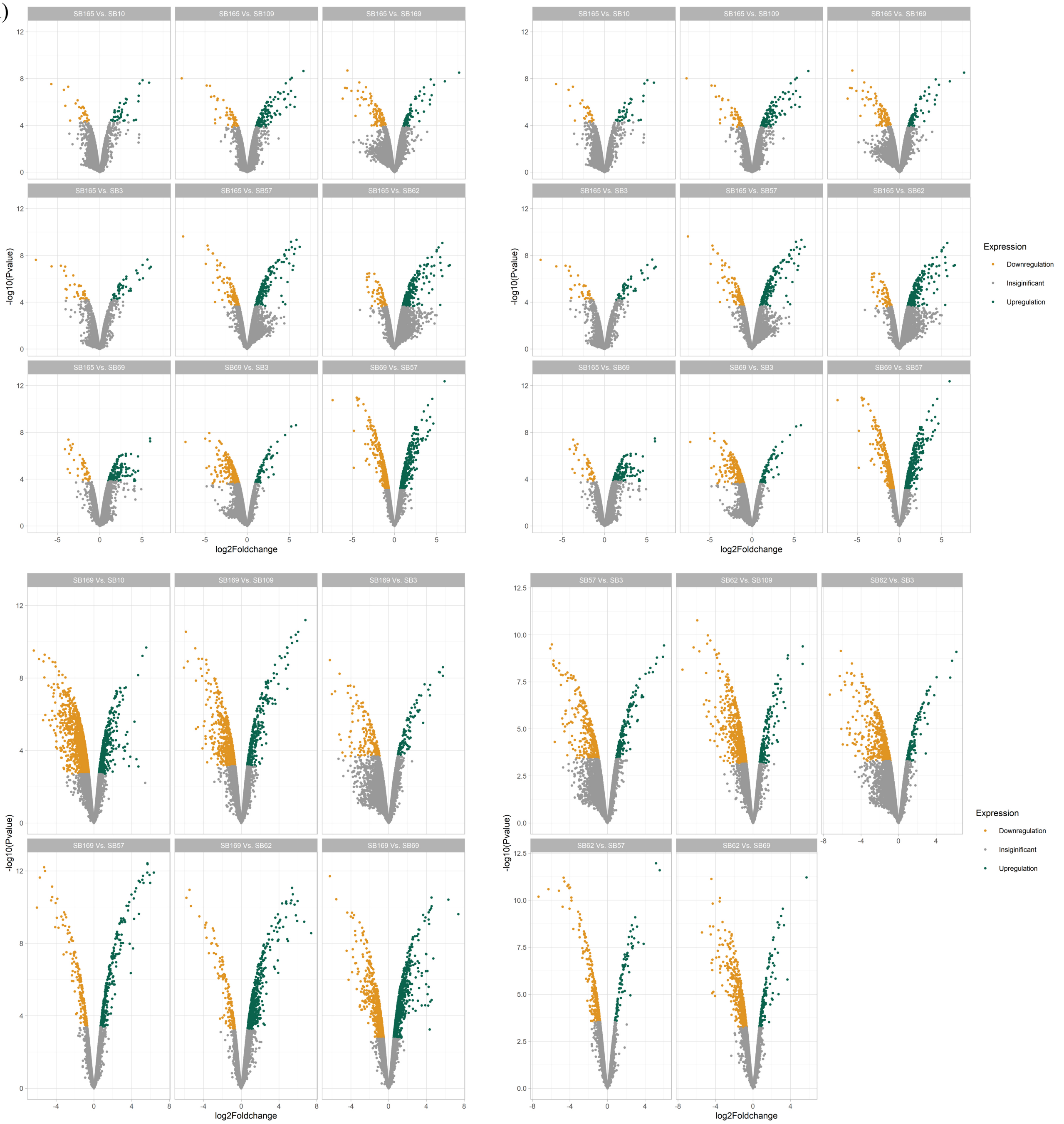
SB165 Vs. SB10
SB165 Vs. SB109
SB165 Vs. SB169
SB165 Vs. SB3
SB165 Vs. SB57
SB165 Vs. SB62
SB165 Vs. SB69
SB69 Vs. SB3
SB69 Vs. SB57
-log10(Pvalue)
log2Foldchange
Expression
Downregulation
Insiginificant
Upregulation
SB169 Vs. SB10
SB169 Vs. SB109
SB169 Vs. SB3
SB169 Vs. SB57
SB169 Vs. SB62
SB169 Vs. SB69
SB57 Vs. SB3
SB62 Vs. SB109
SB62 Vs. SB3
SB62 Vs. SB57
SB62 Vs. SB69

(B)

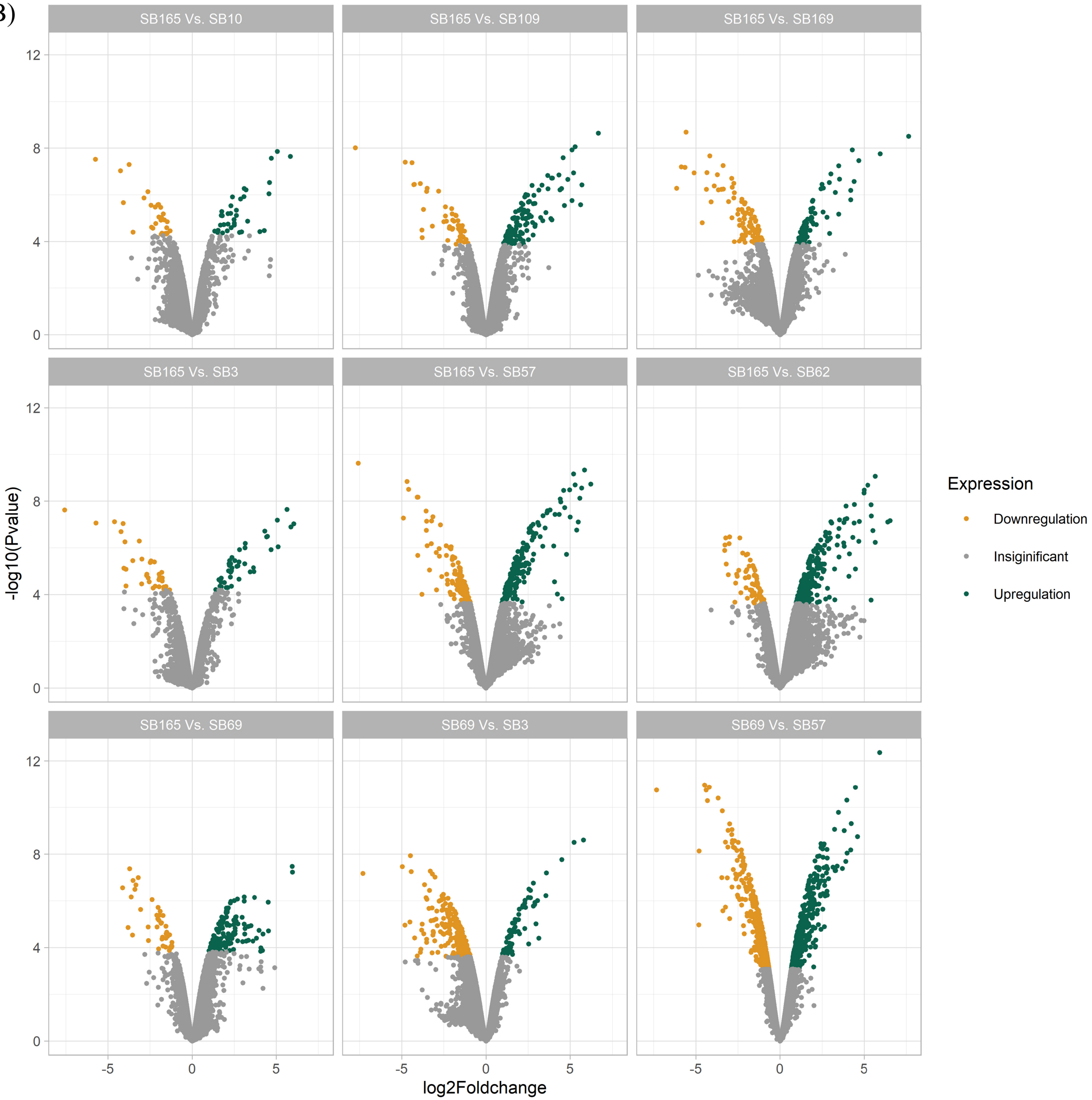

Figure S2: The volcano plots showing differentially expressed genes (DEGs) in the tissue of (A) peduncle of eight lines of Seri/Bebax contrasting for total stem WSCs and treated with the rainfed environment and (B) flag leaf of WL-711 (high yielding drought-sensitive) and C-306 (high yielding drought tolerant) genotypes treated with well-watered and complete drought stress environments. The negative log10 transformed FDR values test the null hypothesis of no difference in expression levels between high and low wheat genotype (y-axis) and are plotted against the average log fold changes (FC) in expression (x-axis). Insignificant DEGs are plotted in grey. In orange and green are significant ($p \geq 0.05$) DEGs and represented as down- and up-regulated genes, respectively.

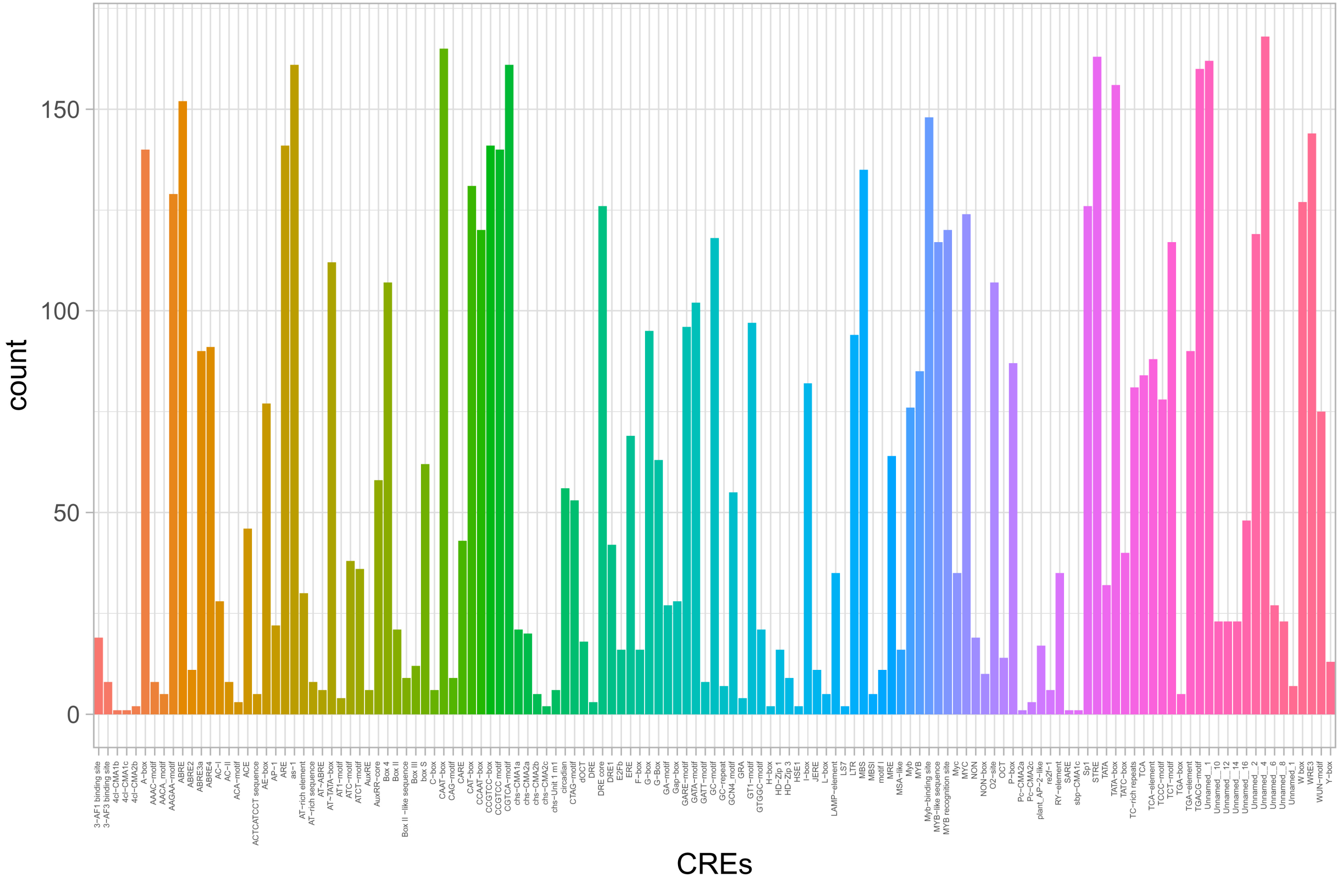

Figure S3: Occurrence of *cis*-regulatory elements identified in 1.5Kbpp upstream sequence of 150 overlapping genes.

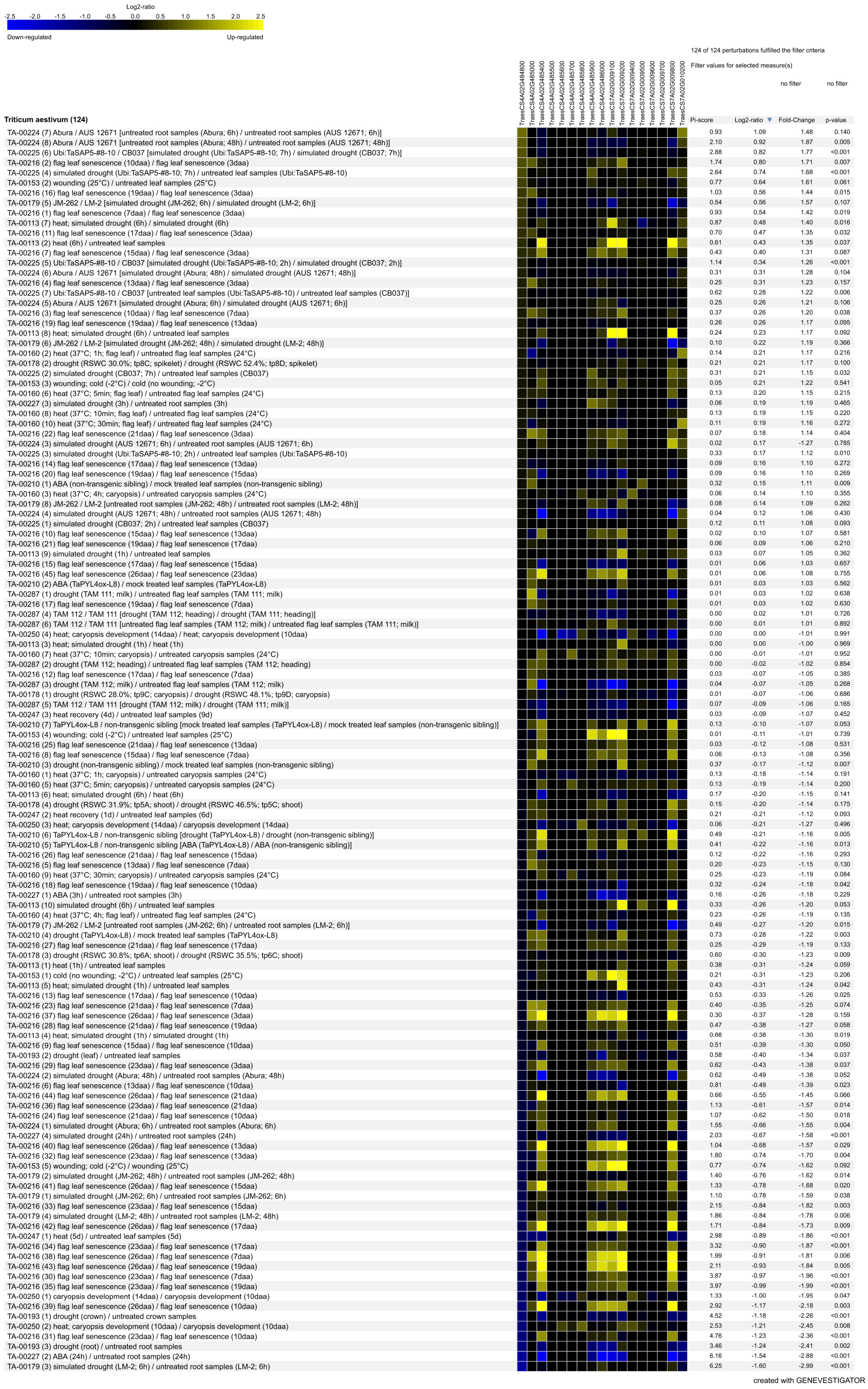

Figure S4: mRNA based differential expression of fructans metabolic genes reported in the QTNs region *qWSC-4A.2* and *qWSC-7A.2* at 124 drought and related perturbations in various samples of *Triticum aestivum* as retrieved from Genevestigator

(A)

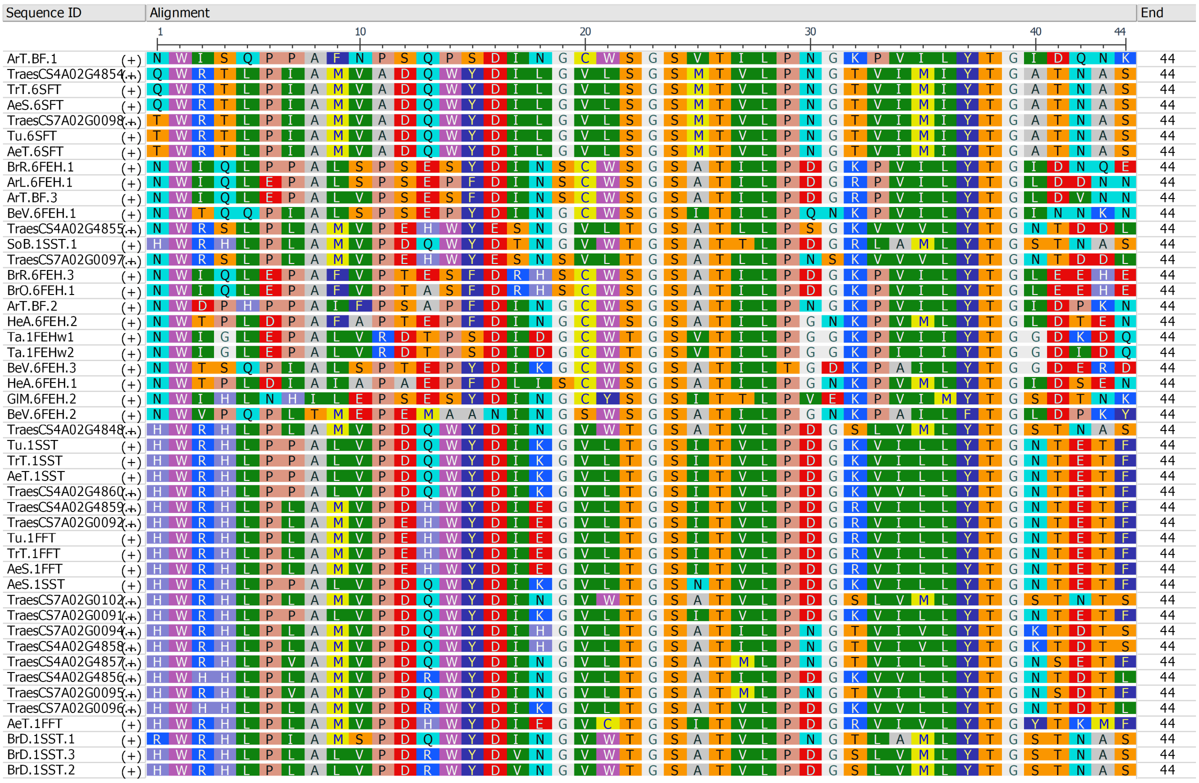
Sequence ID
Alignment
End
1
10
20
30
40
44
ArT.BF.1
TraesCS4A02G4854
TrT.6SFT
AeS.6SFT
TraesCS7A02G0098
Tu.6SFT
AeT.6SFT
BrR.6FEH.1
ArL.6FEH.1
ArT.BF.3
BeV.6FEH.1
TraesCS4A02G4855
SoB.1SST.1
TraesCS7A02G0097
BrR.6FEH.3
BrO.6FEH.1
ArT.BF.2
HeA.6FEH.2
Ta.1FEHw1
Ta.1FEHw2
BeV.6FEH.3
HeA.6FEH.1
GlM.6FEH.2
BeV.6FEH.2
TraesCS4A02G4848
Tu.1SST
TrT.1SST
AeT.1SST
TraesCS4A02G4860
TraesCS4A02G4859
TraesCS7A02G0092
Tu.1FFT
TrT.1FFT
AeS.1FFT
AeS.1SST
TraesCS7A02G0102
TraesCS7A02G0091
TraesCS7A02G0094
TraesCS4A02G4858
TraesCS4A02G4857
TraesCS4A02G4856
TraesCS7A02G0095
TraesCS7A02G0096
AeT.1FFT
BrD.1SST.1
BrD.1SST.3
BrD.1SST.2

(B)

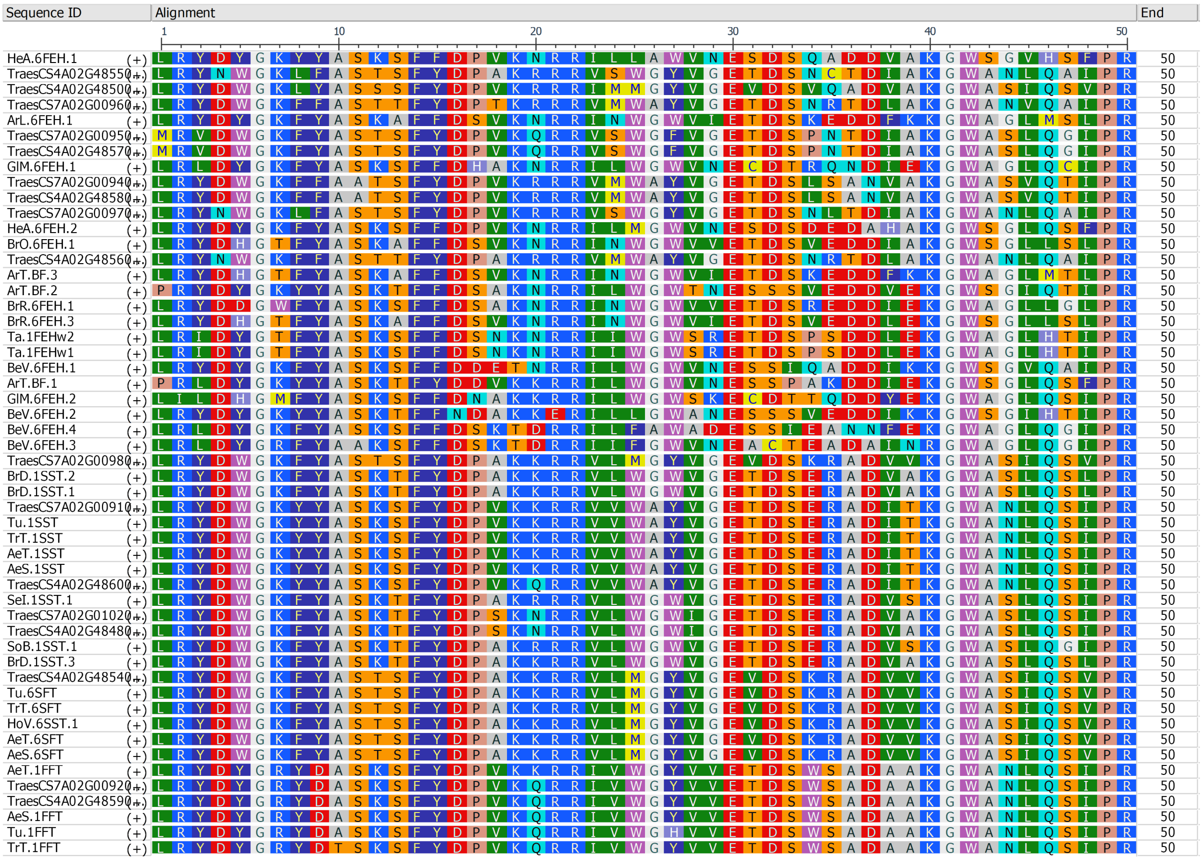
Sequence ID
Alignment
End
1
10
20
30
40
50
HeA.6FEH.1
TraesCS4A02G48550
TraesCS4A02G48500
TraesCS7A02G00960
ArL.6FEH.1
TraesCS7A02G00950
TraesCS4A02G48570
GlM.6FEH.1
TraesCS7A02G00940
TraesCS4A02G48580
TraesCS7A02G00970
HeA.6FEH.2
BrO.6FEH.1
TraesCS4A02G48560
ArT.BF.3
ArT.BF.2
BrR.6FEH.1
BrR.6FEH.3
Ta.1FEHw2
Ta.1FEHw1
BeV.6FEH.1
ArT.BF.1
GlM.6FEH.2
BeV.6FEH.2
BeV.6FEH.4
BeV.6FEH.3
TraesCS7A02G00980
BrD.1SST.2
BrD.1SST.1
TraesCS7A02G00910
Tu.1SST
TrT.1SST
AeT.1SST
AeS.1SST
TraesCS4A02G48600
SeI.1SST.1
TraesCS7A02G01020
TraesCS4A02G48480
SoB.1SST.1
BrD.1SST.3
TraesCS4A02G48540
Tu.6SFT
TrT.6SFT
HoV.6SST.1
AeT.6SFT
AeS.6SFT
AeT.1FFT
TraesCS7A02G00920
TraesCS4A02G48590
AeS.1FFT
Tu.1FFT
TrT.1FFT

(C)

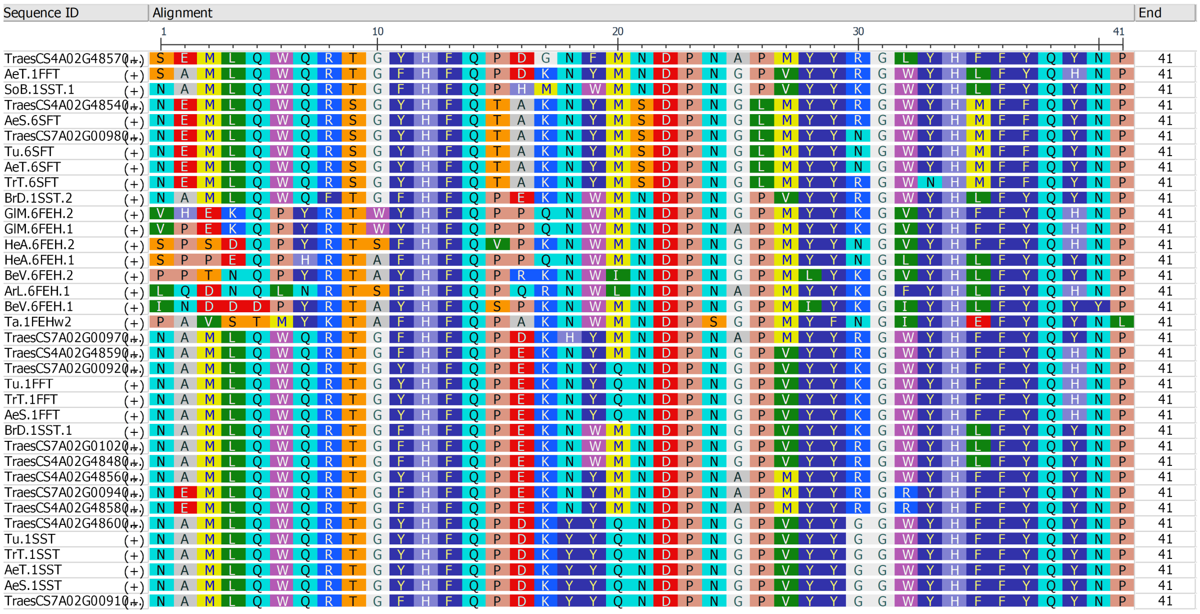

Figure S5: Multiple sequnce alignment of motifs (A) Glyco hydro 32N, (B) Glyco hydro32C and (C) INV N Identified in fructans metabolic genes in model species and *qWSC-4A.2* and *qWSC-7A.2* in *Triticum aestivum*

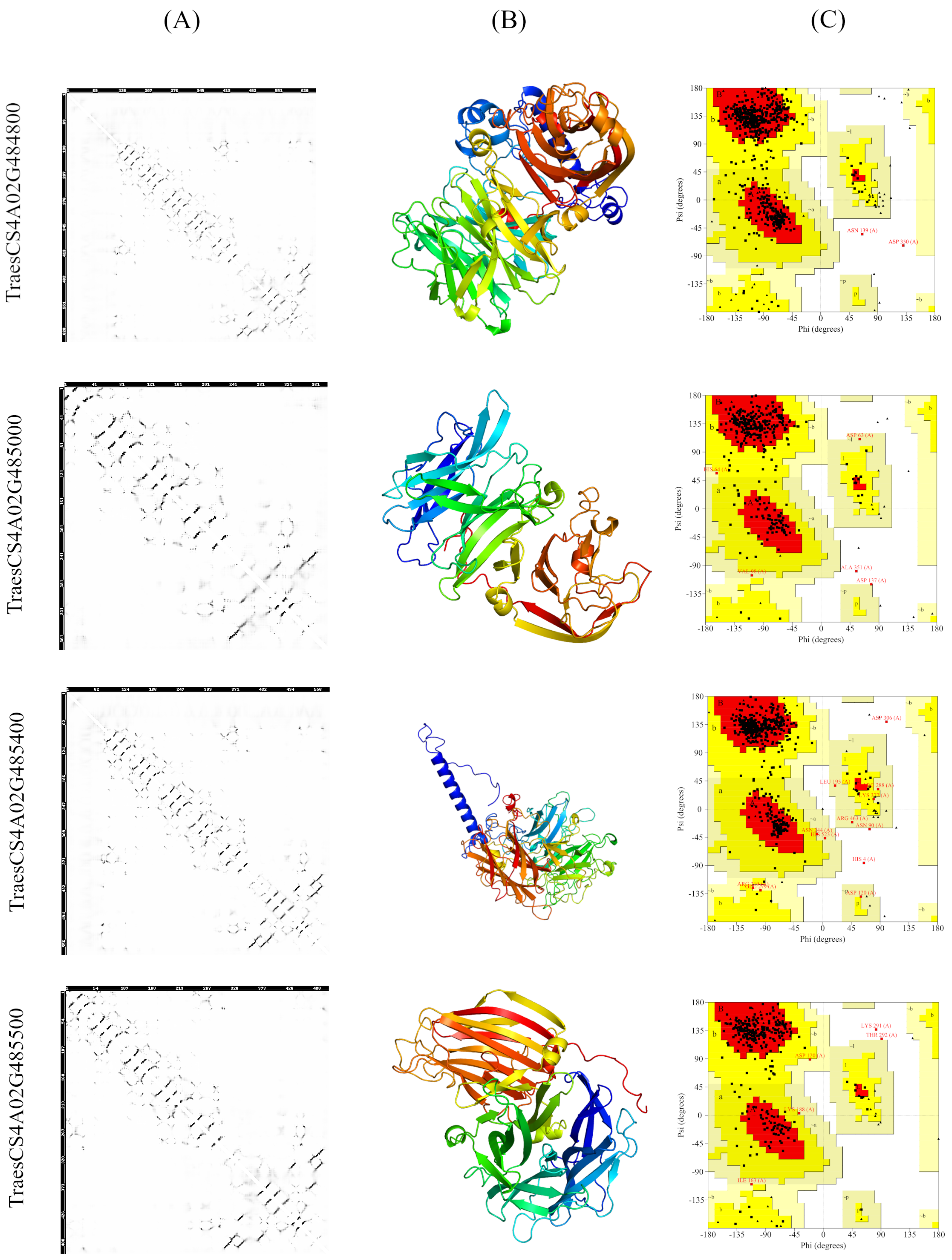
(A)
(B)
(C)
TraesCS4A02G484800
TraesCS4A02G485000
TraesCS4A02G485400
TraesCS4A02G485500
Psi (degrees)
Phi (degrees)

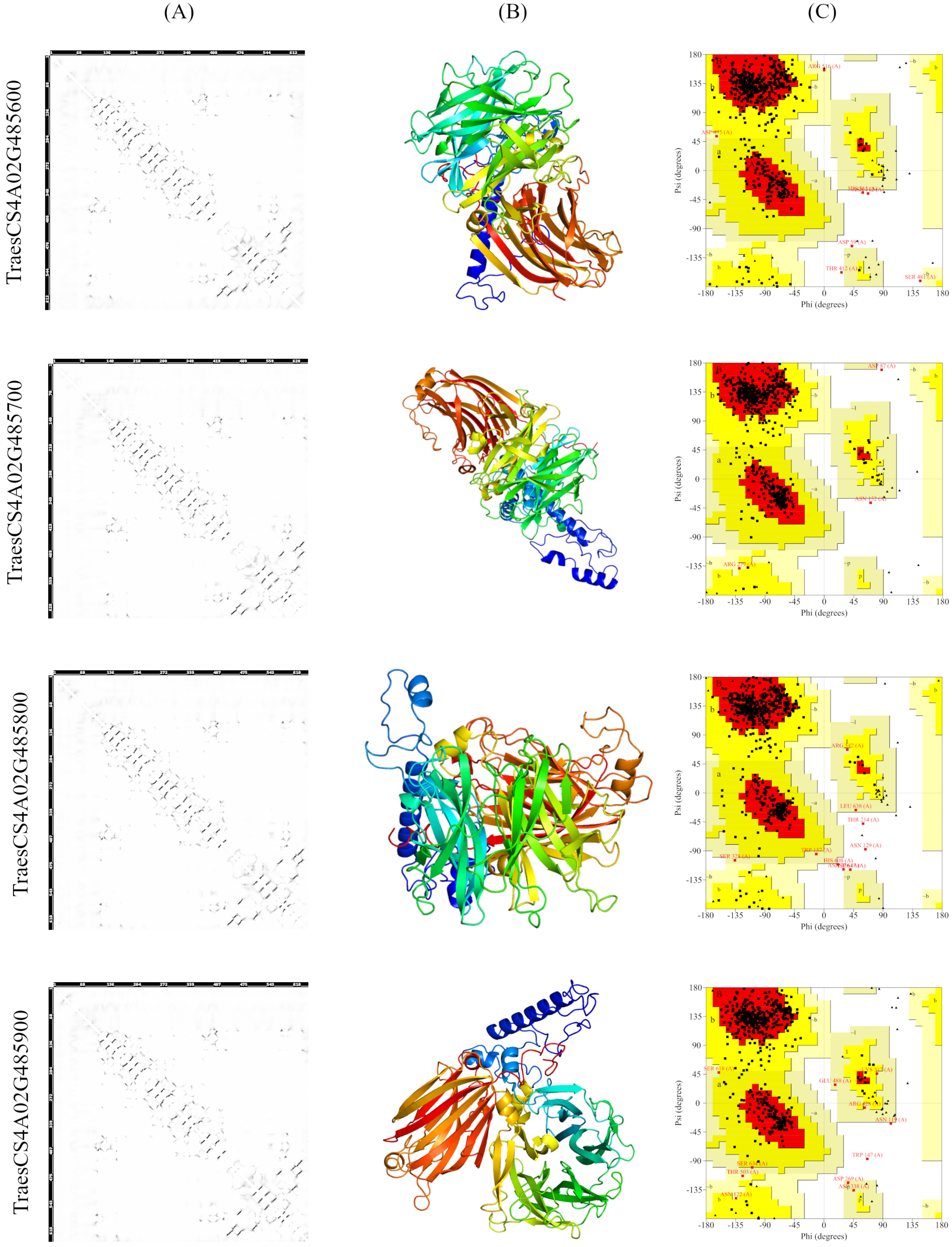
(A)
(B)
(C)
TraesCS4A02G485600
TraesCS4A02G485700
TraesCS4A02G485800
TraesCS4A02G485900
Psi (degrees)
Phi (degrees)

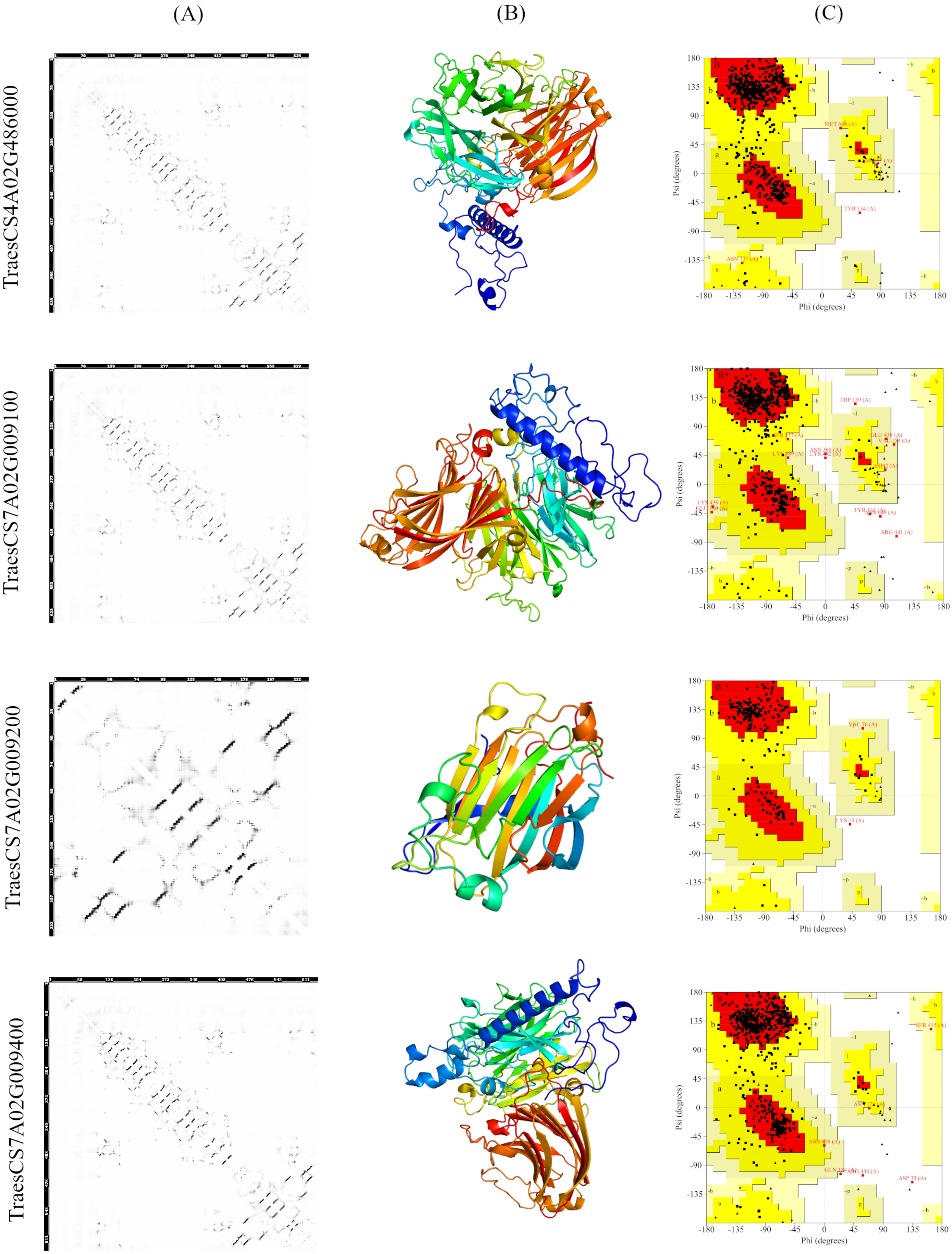
(A)
(B)
(C)
TraesCS4A02G486000
TraesCS7A02G009100
TraesCS7A02G009200
TraesCS7A02G009400
Psi (degrees)
Phi (degrees)

(A) (B) (C)

TraesCS7A02G009500

TraesCS7A02G009600

TraesCS7A02G009700

TraesCS7A02G009800

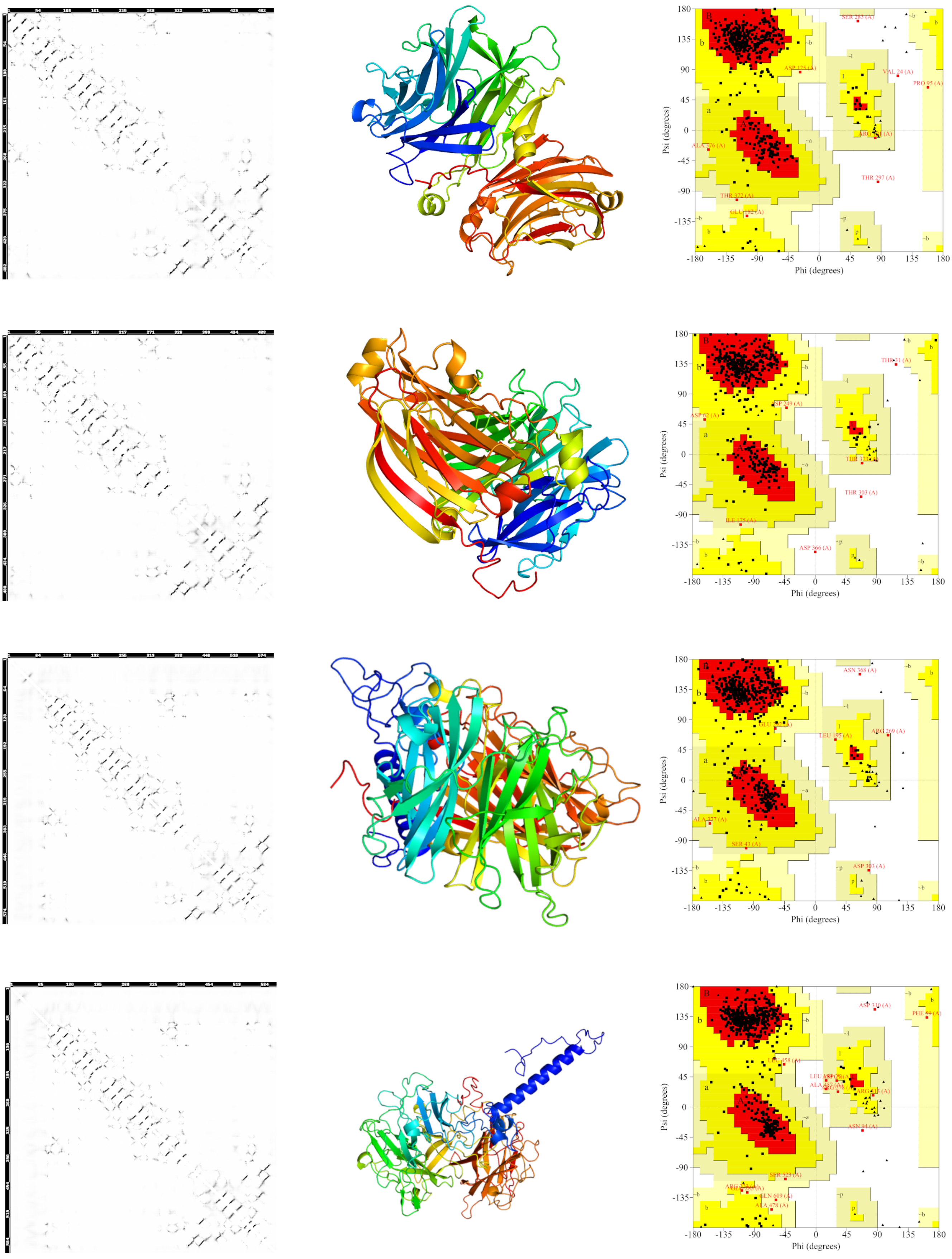

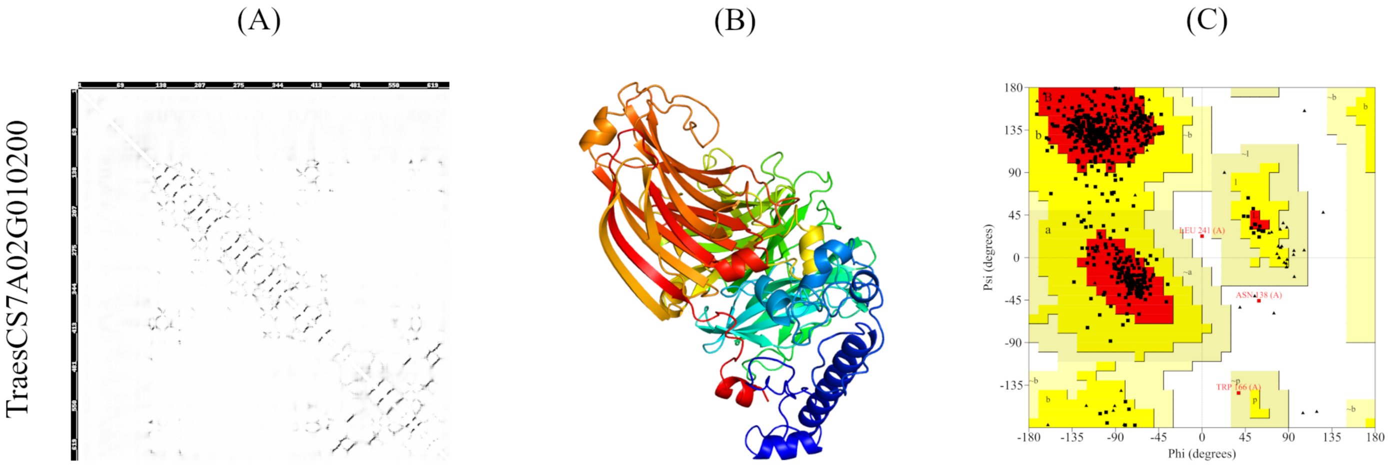

Figure S6: Structural differences in the tertiary structure of the proteins translated by different fructans metabolic genes reported in the quantitative nucleotide regions *qWSC-4A.2* and *qWSC-7A.2*. The structures are predicted with contact and distance-based protein folding powered by DeepLearning as implicated in the RaptoraX web server. (A) shows the predicted contact probability matrix. A contact is defined by Cb-Cb distance <=8Å. Darker color indicates a higher probability. (B) offers the 3D protein models generated in ChimeraX with resulting pdb files. (C) shows the Ramachandran plots showing amino acid placement in allowed and disallowed regions
