# Supplementary material for: GWAS to Identify Novel QTNs for WSCs Accumulation in Wheat Peduncle Under Different Water Regimes
Source: Front Plant Sci. 2022 Mar 3;13:825687. doi: 10.3389/fpls.2022.825687 (PMC8928439; doi:10.3389/fpls.2022.825687)
Supplement: Supplementary file 2 [file Data_Sheet_2.PDF]

Supplementary methods

Method M1: Gene prioritisation

The list of candidate genes was narrowed down to high priority candidate genes by evaluating them on three candidate gene prioritisation approaches *viz*. 1) knowledge-based gene prioritisation (KGP), 2) differential expression based gene prioritization (DEGP) and, 3) co-regulatory network-based gene prioritization (CNGP).

For KGP, the Knetminer tool was used. Genes were searched keywords 'fructose', 'glucose', 'sucrose', 'fructans', 'WSC' and 'water soluble carbohydrates. A local database was developed with a list of all the retrieved genes. Genes with significant evidences ($p<0.005$) tfalling within 1Mbp region of significant QTNs were prioritised.

For DEG, the CEL files of publicly Affymetrix data of GSE9767 (Xue *et al.*, 2008) and GSE87325 (Kumar *et al.*, 2018) were retrieved from NCBI's Gene Expression Omnibus (GEO) database. Differentially expressed genes (DEGs) between all possible sample combinations were identified by analysing the Affymetrix data using GEO2R tool. The *p*-value adjustment (corrected $p \leq 0.01$) was done using the Benjamini Hochberg false discovery rate (FDR) with multiple testing correction algorithms. The significance cut-off level for DEGs was set at $p \leq 0.05$. For visualisation DEGs volcano plots were developed in R using *ggplot2* package, only with the genes falling within the 1Mbp window of significant QTNs.

For developing co-regulatory networks, the MAS5 normalised intensities of probe sets were analysed using GeneSpring GX v12 software (Agilent Technologies, Santa Clara, USA). Genes within 1Mbp window of significant QTNs were filtered and used to develop GLASSO-SF co-regulatory networks at λ =0.07 in GeNeCK webserver (Zhang *et al.*, 2019). Genes with top 10% connections were identified as hub genes with cytoHubba (Chin *et al.*, 2014) app in Cytoscape and were prioritised.

Genes equally prioritised by all three approaches were called high priority candidate genes (HPCGs), whereas genes prioritised by DEGP and CNGP were called low priority candidate genes (LPCGs). Overlapping genes were found in either coregulatory network and called specially prioritised genes (SPCG).

Method M2: Phylogenetic and protein structure study of fructans metabolic genes

Amino acid sequences (AAS) of fructans metabolic genes from 15 model species and Triticum aestivum from *qWSC*-4A.2 and *qWSC*-7A.2 (Supplementary Table S11) were retrieved from EnsemblPlants and NCBI. Multiple sequence alignments were carried with *ClustalW* followed by constructing a Maximum Likelihood tree with 10^3 bootstraps in the MEGA-X tool (Kumar *et al.*,

2018). Motifs in the AASs were identified with the MEME suite 5.4.1 (Bailey *et al.*, 2015) and interactively visualised with iTOL web server.

Physical properties of AASs were identified with Expasy's ProtParm web server. Furthermore, structures of proteins translated by fructans metabolic genes reported in *qWSC*-4A.2 and *qWSC*-7A.2 were predicted by contact and distance-based protein folding algorithms powered by deep learning (Wang and Xu, 2013; Ma *et al.*, 2015) as implicated in RaptorX web server (Källberg *et al.*, 2012). Furthermore, the 3D protein structures were validated based on the ERRAT (Colovos and Yeates, 1993), Verify3D (Lüthy *et al.*, 1992) and the PROCHECK (Laskowski *et al.*, 1993) with Structure Validation Server (SAVES).

References

Bailey, T. L., Johnson, J., Grant, C. E., and Noble, W. S. (2015). The MEME suite. *Nucleic Acids Res.*, *43*(W1), W39-W49.

Chin, C. H., Chen, S. H., Wu, H. H., Ho, C. W., Ko, M. T., and Lin, C. Y. (2014). cytoHubba: identifying hub objects and sub-networks from complex interactome. *BMC Syst. Biol.*, *8*(4), 1-7.

Colovos, C., and Yeates, T. O. (1993). Verification of protein structures: patterns of nonbonded atomic interactions. *Protein Sci.*, *2*(9), 1511-1519.

Källberg, M., Wang, H., Wang, S., Peng, J., Wang, Z., Lu, H., and Xu, J. (2012). Template-based protein structure modeling using the RaptorX web server. *Nat. Protoc.*, *7*(8), 1511-1522.

Kumar, S., Stecher, G., Li, M., Knyaz, C., and Tamura, K. (2018). MEGA X: molecular evolutionary genetics analysis across computing platforms. *Mol. Biol. Evol.*, *35*(6), 1547.

Laskowski, R. A., MacArthur, M. W., Moss, D. S., and Thornton, J. M. (1993). PROCHECK - a program to check the stereochemical quality of protein structures. *J. App. Cryst.*, 26, 283-291.

Lüthy, R., Bowie, J. U., and Eisenberg, D. (1992). Assessment of protein models with three-dimensional profiles. *Nature*, *356*(6364), 83-85.

Ma, J., Wang, S., Wang, Z., and Xu, J. (2015). Protein contact prediction by integrating joint evolutionary coupling analysis and supervised learning. *Bioinformatics*, *31*(21), 3506-3513.

Zhang, M., Li, Q., Yu, D., Yao, B., Guo, W., Xie, Y., and Xiao, G. (2019). GeNeCK: a web server for gene network construction and visualization. *BMC Bioinform.*, *20*(1), 1-7.

Wang, Z., and Xu, J. (2013). Predicting protein contact map using evolutionary and physical constraints by integer programming. *Bioinformatics*, *29*(13), i266-i273.
